# Nanomaterials for Energy Storage Systems—A Review

**DOI:** 10.3390/molecules30040883

**Published:** 2025-02-14

**Authors:** Habeeb Mohammed, Md Farouq Mia, Jasmine Wiggins, Salil Desai

**Affiliations:** 1 Department of Industrial and Systems Engineering, North Carolina Agricultural and Technical State University, Greensboro, NC 27411, USA; mmohammed@aggies.ncat.edu (H.M.); jmwiggins@aggies.ncat.edu (J.W.); 2 Department of Applied Engineering and Technology, North Carolina Agricultural and Technical State University, Greensboro, NC 27411, USA; mmia@aggies.ncat.edu; 3 Center of Excellence in Product Design and Advanced Manufacturing, North Carolina Agricultural and Technical State University, Greensboro, NC 27411, USA

**Keywords:** energy storage, lithium-ion, sodium–sulfur, redox flow, supercapacitors, nanomaterials

## Abstract

The ever-increasing global energy demand necessitates the development of efficient, sustainable, and high-performance energy storage systems. Nanotechnology, through the manipulation of materials at the nanoscale, offers significant potential for enhancing the performance of energy storage devices due to unique properties such as increased surface area and improved conductivity. This review paper investigates the crucial role of nanotechnology in advancing energy storage technologies, with a specific focus on capacitors and batteries, including lithium-ion, sodium–sulfur, and redox flow. We explore the diverse applications of nanomaterials in batteries, encompassing electrode materials (e.g., carbon nanotubes, metal oxides), electrolytes, and separators. To address challenges like interfacial side reactions, advanced nanostructured materials are being developed. We also delve into various manufacturing methods for nanomaterials, including top–down (e.g., ball milling), bottom–up (e.g., chemical vapor deposition), and hybrid approaches, highlighting their scalability considerations. While challenges such as cost-effectiveness and environmental concerns persist, the outlook for nanotechnology in energy storage remains promising, with emerging trends including solid-state batteries and the integration of nanomaterials with artificial intelligence for optimized energy storage.

## 1. Introduction

As our population expands and economies develop, improved efficiency and sustainability in energy storage solutions become increasingly important. With the rise of renewable sources like solar and wind, which are not consistently available, maintaining grid stability and reliability poses a significant challenge. Thus, progressing energy storage technology is essential for incorporating renewables into our energy systems and securing a sustainable future. According to Kumar et al. [1], over the past four decades, energy demand has almost mirrored GDP growth. Historically, a 1% increase in global GDP has led to a 0.6% rise in primary energy consumption, projecting an annual energy demand growth rate of around 2% in the future. The authors predict that by 2030, the demand for energy storage will be four times what it is today, requiring specialized equipment and systems to handle the expected energy needs effectively. Energy storage is crucial for ensuring energy security and reliable supply. As the need for energy storage solutions grows, there is a corresponding demand for research into various technologies and their applications, contributing to sustainable power storage. Integrating renewable energy sources (RES) into existing energy systems is challenging due to their variability [2]. Therefore, adequate energy storage is essential for managing the intermittent nature of renewable energy, maximizing RES benefits, and reducing overall carbon footprints.

Nanotechnology significantly enhances energy storage systems through various mechanisms like increased surface area, improved charge transport, and electrode stability [3]. Nanomaterials—such as nanowires, nanotubes, and nanoparticles—are larger in terms of surface area than similar kinds of materials. This provides more active sites for energy storage reactions, resulting in higher energy densities as well as faster rates of charging and discharging [3]. The unique properties of nanomaterials also improve charge transport within energy storage devices, boosting the efficiency and performance of batteries and supercapacitors [4]. Graphene-based materials and other nanomaterials have emerged as favorable alternatives for energy storage devices, thanks to their large surface area and excellent electrical conductivity. These features enable the development of high-capacity, fast-charging batteries, which are crucial for applications like electric vehicles and grid-scale energy storage [3]. Nanotechnology also advances the United Nations Sustainable Development Goals (SDGs) through innovative applications, including energy storage systems. It offers cleaner and more sustainable energy storage solutions by ensuring improved conversion processes and enhanced efficiency [5].

This review paper comprehensively examines the latest advancements in nanotechnology for energy storage applications, with a particular focus on batteries and capacitors. We delve into the various ways nanomaterials are being integrated into different energy storage systems, including a range of battery technologies such as lithium-ion batteries (LiBs), sodium–sulfur (Na-S) batteries, and redox flow batteries. We analyze how nanomaterials are being utilized to improve the performance of key components like electrodes, electrolytes, and separators. In addition, we explore the use of nanomaterials in enhancing the energy storage capacity and efficiency of supercapacitors. While we touch upon various battery technologies, this review dedicates significant attention to LiBs due to their widespread adoption in electric vehicles (EVs), grid-scale energy storage, and most portable electronics.

**Figure 1:**
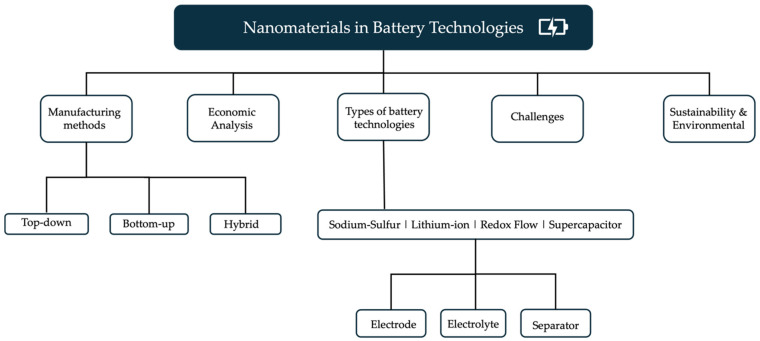
Schematic diagram of the outline of the review paper.

Furthermore, this paper examines the common manufacturing processes employed in the production of these modern energy storage devices, particularly those utilizing nanostructured materials. We also address the challenges and potential solutions associated with incorporating nanomaterials into energy systems, including issues such as stability, scalability, and safety concerns. An economic analysis is presented, outlining the costs associated with developing and deploying these cutting-edge energy storage technologies. Finally, this paper unravels the sustainability and environmental implications of utilizing nanomaterials in battery production and their potential impact on the environment. Figure 1 presents a visual representation outlining the structure of this review paper.

The comprehensive scope and detailed outline of this review paper aim to provide a valuable resource for researchers, engineers, and policymakers seeking to stay abreast of the latest advancements in nanotechnology-based energy storage. By thoroughly analyzing the current state-of-the-art in nanomaterial-enhanced battery systems, this paper not only identifies promising avenues for future research but also suggests potential solutions to address the existing challenges in this critical domain. Moreover, the insights gained from this work can be invaluable for policymakers and decision-makers, enabling them to make informed decisions regarding research funding, regulatory policies, and market incentives that can foster the continued advancement and responsible implementation of these transformative technologies. This review paper seeks to bridge the gap between fundamental research and real-world applications, ultimately accelerating the development of next-generation energy storage solutions that are both sustainable and efficient.

## 2. Nanotechnology and Nanomaterials

**Figure 2:**
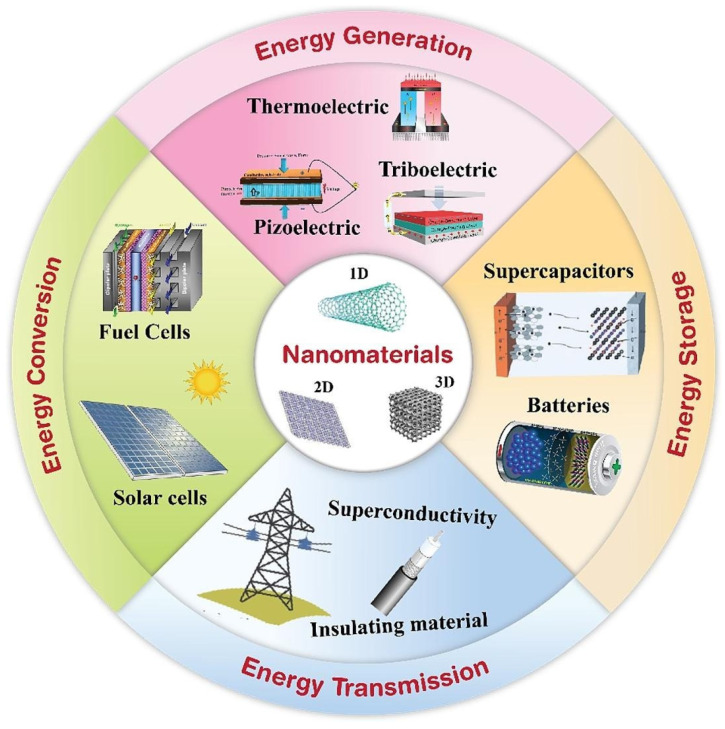
Schematic demonstration of various nanomaterial applications in different areas [14]. © Elsevier, 2024.

The United States National Nanotechnology Initiative denotes nanotechnology as the manipulation of materials that are within a scale between 1 and 100 nanometers [6]. Recent nanotechnology research is concentrated on bottom–up, top–down, and hybrid approaches [7,8,9,10,11,12,13]. Figure 2 shows the applications of nanomaterials in different fields.

**Figure 3:**
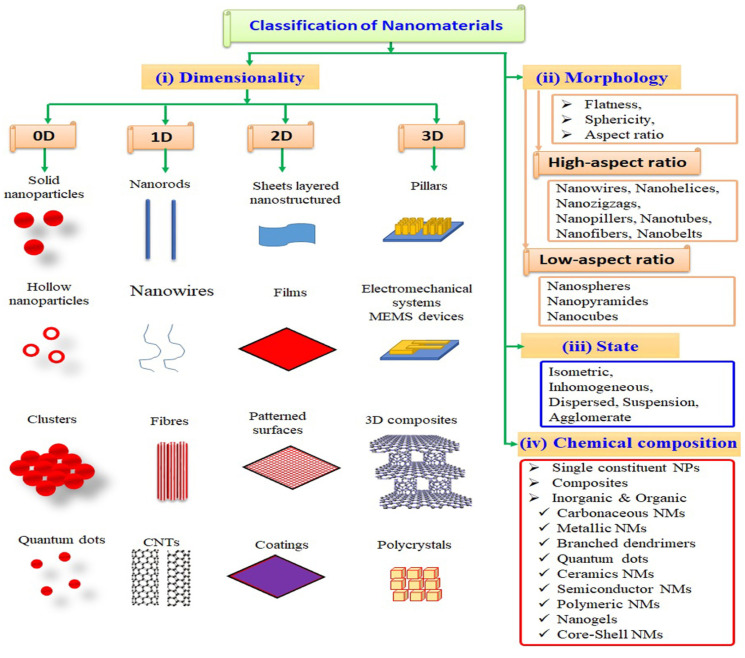
Classification of nanomaterials [18]. © Elsevier, 2020.

Interestingly, materials may display peculiar characteristics that are very different from those of mesoscale materials [15,16,17]. Chemical composition is important in the classification of nanomaterials. Compared to mesoscale materials, nanomaterials display unique chemical, visual (optical), and electrical characteristics. For instance, gold (Au) nanoparticles (NPs) can be synthesized by the combination of sono-electrochemical and ultrasonic vibration. Carbon-related nanoscale nanomaterials exhibit a wide range of physical and chemical characteristics in their field of application, which makes them more attractive. The various classes of nanomaterials are shown in Figure 3.

## 3. Nanomaterials in Battery Technologies

### 3.1. Nanotechnology Application in Lithium-Ion Batteries (LiBs)

**Figure 4:**
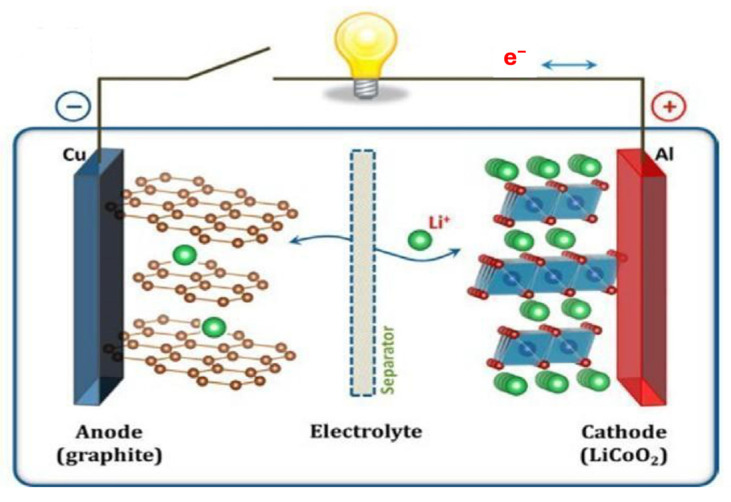
Mode of operation of LiBs. © ACS, 2013 [20].

Li-ion batteries are vital in modern rechargeable batteries. Generally, current cathode materials used include LiCoO_2_, LiMn_2_O_4_, LiFePO_4_, and LiZO_2_, while graphite is used as anode. Moreover, LiPF_6_ salt is dissolved into one or more carbonate-based solvents for an ionic conductor solution called an electrolyte. Polypropylene (PP) is employed as a separator for restricting electron flow. The current collector (aluminum) is used on the positive electrode side, while copper is used on the negative electrode side. LiBs have more advantages, like high charging/discharging capacity, high life cycles, and ease of carrying (compared to other batteries like lead acid and Ni-based batteries). However, the higher energy densities of LiBs are limited (specific capacity 372 mAh/g for graphite anode as well as 100–400 mAh/g for oxide cathode) to meet the current demand of the world [19]. A LiBs system is shown in Figure 4, where Li-ions can transmit between the anode and cathode through the electrolyte and separator.

#### 3.1.1. Negative Electrodes

**Figure 5:**
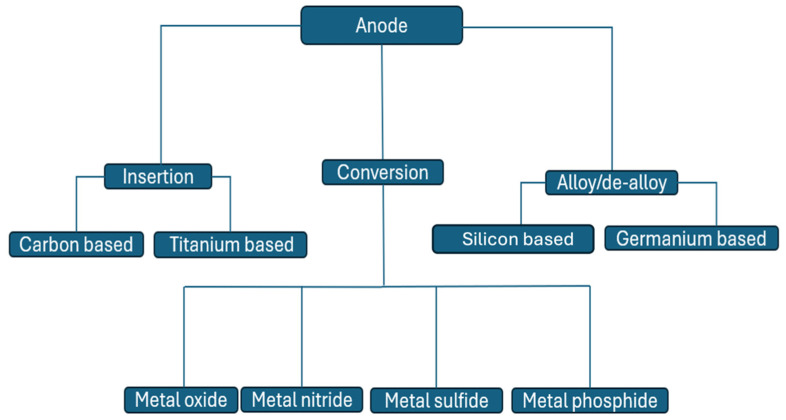
Classification of negative electrode materials.

**Figure 6:**
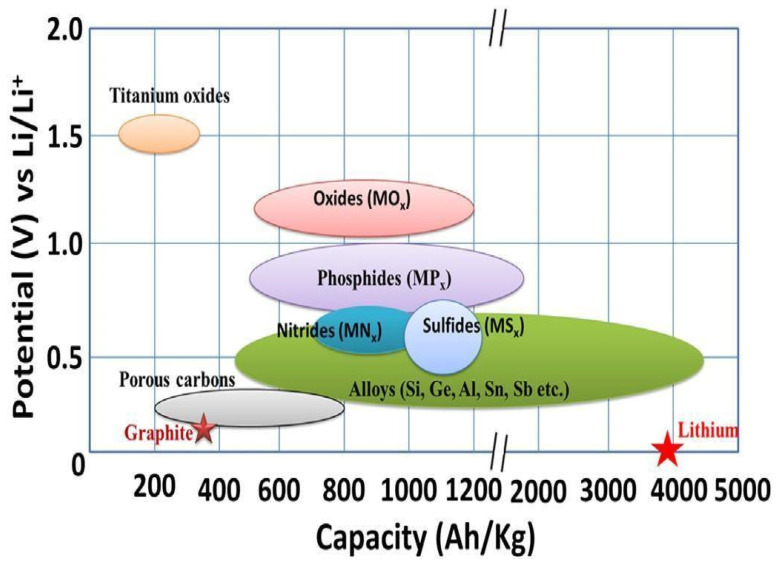
Relationship between capacity and potential voltage of different types of anode materials. © Elsevier, 2014 [23].

Negative electrode/anode materials play an important role in the various components of LiBs. The nanomaterials in the anode of LiBs are vital for fostering lithium intercalation and deintercalation more than bulk materials, resulting in improved capacity and high electron collection efficiency [21,22]. In Figure 5, three types of nanomaterial anodes of LiBs are illustrated. Also, insertion materials come in two forms: carbon-based and titanium based. Conversion materials are metals with oxides, sulfides, phosphides, and nitrides. Alloy materials are generally silicon, carbon, or germanium based. Figure 5 and Figure 6 show the various classes of anode materials and their potential voltages, while Table 1 compares the negative electrode nanomaterials used in LiBs.

##### Insertion

Intercalation and deintercalation materials are carbon and titanium based. Carbon-based material is employed widely as anode material in LiBs. This is because carbon-based materials have several properties, such as significant reversible cycles, chemical stability, electrochemical stability, thermal condition, ease of availability, low cost, among others [28]. Zhang et al. [29] systematically fabricated carbon coating for anode in LiBs. They justified the carbon-coated and non-carbon coated anode performance with solid electrolyte interphase (SEI) formation around the circumstance of the anode. They figured out that the coated anode improved the performance. This is because the coated anode’s SEI film was thin and dense (60 to 150 nm thickness) compared to the uncoated anode (450 to 980 nm thickness). Finally, the authors reported that carbon coating would reduce the electrolyte’s decomposition due to inducing thin SEI-layer formation on the anode.

Furthermore, titanium-based materials are vital in LiBs. This is because it has minor safety issues, which helps with better design, low cost, less toxicity, little volume change, particularly 2 to 3% on both intercalation and deintercalation, and more cycle life, among others [30,31,32,33]. On the other hand, it has some drawbacks, like low conductivity and low theoretical capacity, especially from 175 to 330 mAh/g. Further study is required to overcome the difficulties. Another point worth noting is that the titanium-based oxide facilitates electrochemical performance and lithium insertion ability with their size, form or morphology, and structure. More importantly, nanostructured titanium oxides are better than bulk materials because of their relatively higher capacity and longer life cycle [34,35].

i. Carbon nanotubes (CNTs)

CNTs are rolled graphene sheets with one or more layers [36,37]. A variety of methods, including ball milling, acid oxidation, and chemical vapor deposition, are used to create carbon nanotubes (CNTs), which are then used as negative electrode materials for LiBs [22]. CNTs can pose a strain on their hexagonal lattice structure due to their smaller diameter, resulting in a strong graphitic sheet. Hence, such properties make them electronegative. Hence, CNTs are more preferred in anode applications. One layer sheet is termed single wall carbon nanotubes (SWCNTs), while more layer sheets are termed multi wall carbon nanotubes (MWCNTs) [38,39]. CNT electrode is better than a traditional graphite electrode because it has more capacity for energy storage and conversion. Hence, such electrodes have more conductivity and stability. According to studies, both sides (internal and external) of CNTs can absorb Li-ion, but the interior side absorbs more lithium than the external side of CNTs. Also, the length of CNTs is a factor because shorter nanotubes assist in inserting/de-inserting Li-ion, but lengthy nanotubes obstruct Li-ion diffusion [40].

**Figure 7:**
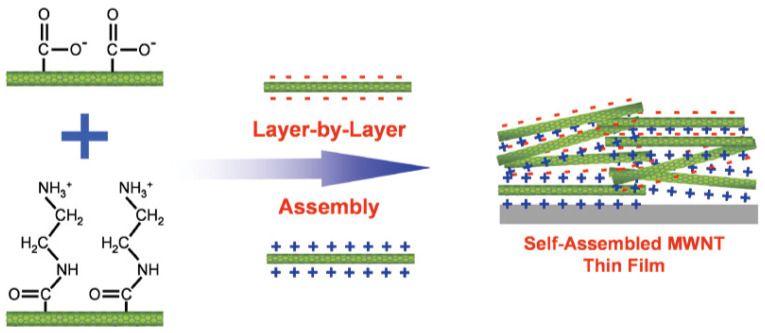
Self-assembled MWNT thin film with charged MWNTs [46]. © ACS, 2009.

With respect to formation, there are two categories of CNT electrodes: entangled random network (ECNT) and an array structure (ACNT). The preparation of ECNT-based electrodes through the traditional process involves dispersing CNTs equally on the current collector, then blending CNTs powder into polyvinylidene fluoride (PVDF) suspension, and then coating it on a metal planar like a Cu, Ni, or Pt sheet. However, in the traditional process, there is very little conductive contact between active materials and substrate. To figure out this issue, the binder-free CNT can be considered via the EPD (electrophoretic deposition) or LBL (layer by layer) deposition method. There are many studies in which EPD methods are employed to achieve a high lithium storage capacity [41,42,43]. As shown in Figure 7, the LBL method is able to precisely control thickness and shape due to the interaction of cations and anions with functional groups affixed to outside walls of CNTs [44,45,46].

ii. Graphene

Graphene plays a vital role in LiBs as anode material because of its conductivity, structural flexibility, higher charge mobility, electrical and mechanical characteristics, lightweight, and good surface region. Lian et al. [47] fabricated high-quality graphene (thin wrinkled paper) with four layers with a specified area (over 490 m^2^/g). The reversible capacity of graphene is 1264 mA/g, with a 100 mA/g current density at the initial cycle and 848 mA/g at the end of the 40th cycle. Yang et al. [48] fabricated a graphene, which can be used as an additive of cathode to the pouch cell. This gives up to 10 Ah current, leading to a reliable evaluation of potential uses in the LiBs. More importantly, graphene additive properties are better compared to commercial additives for better electric conduction.

The theoretical studies on graphene’s lithium storage are very debatable. While a single layer of graphene adsorbed lithium less than that of graphite (372 mAh/g) [49], the performance of graphene surpasses the graphite performance when many sheets of graphene are considered, resulting in either 780 mA/g or 1116 mAh/g [47,48,49,50,51]. Such specific capacities are connected to the two separate interpretations of the synergy with graphene as well as lithium. Specifically, the first one proposes that Li-ions are absorbed on both graphene faces (Li_2_C_6_ stoichiometry), whereas the second one assumes that lithium is confined at the benzene ring in a covalent bond (LiC_2_ stoichiometry).

A recent investigation of LiBs recommends that graphene composites, such as using several combinations of metals–graphene or semiconductors, phosphides–metal, oxides–graphene, and so on, are better as negative electrodes [52,53,54,55]. For instance, it is commonly recognized that SnO_2_ has excellent negative electrode characteristics, but the main disadvantage for a good reversible capacity is its volume changes during cycling. To solve this problem, a graphene–SnO_2_ particle composite was taken into consideration. Indeed, SnO_2_ nanoparticles can be inserted into graphene, which enhances SnO_2_’s electrical conductivity. Specifically, it is suggested that a combination system consist of 2 to 3 nm SnO_2_ particles with nitrogen-doped graphene, which provides gravimetric capacity (1220 mAh/g) after 100 cycles [56].

iii. Spinel Li_4_Ti_5_O_12_ (LTO)

**Figure 8:**
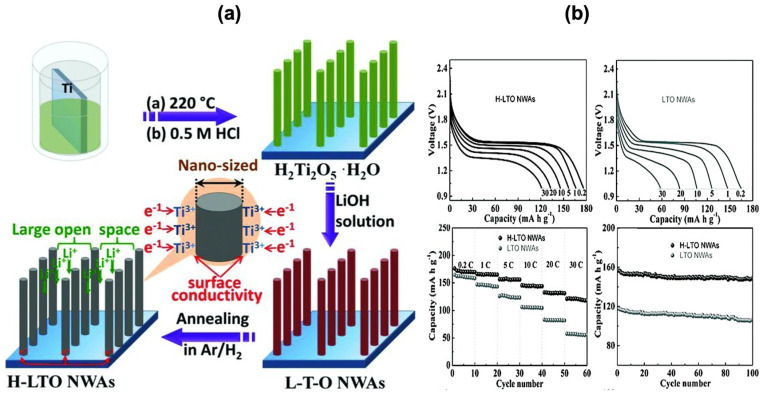
(**a**) Schematic demonstration of hydrogenated LTO fabrication; (**b**) electrochemical performance of LTO and hydrogenated LTO nanowires [57]. © Advanced materials, 2012.

Intercalated titanium-based oxide anode is important in LiBs because it has intrinsic characteristics such as improved safety during cycling, no change in volume, among others. However, low ionic conductivity and very limited theoretical capacity (nearly 175 mAh/g) are major issues for large-scale industrial productions [23]. To address these issues, Shen et al. [57] designed a nanostructured material (LTO-nanowire arrays), which grows on titanium foil for improving ionic conductivity by making Ti^+3^ ions through hydrogenation (see Figure 8). These titanium foils containing nanowires demonstrated excellent rate performance because the theoretical capacity value is close to practical capacity (173 mAh/g at 0.2C rate) with better cycle life when utilized as electrodes directly, without the need for conductive binders or additives. The cycling performance achieved a 95% capacity retention after 100 cycles at 5 °C.

iv. Titanium oxide (TiO_2_)

Titanium oxide plays a vital role in improving properties such as capacity retention, structural stability, high oxidation ability, high chemical steadiness, structural diversity, safety, and low cost of mass production [23,28,58,59,60]. It is also stable at 1.5 V vs. Li/Li^+^ (operating potential). These characteristics make it more promising for anode materials of LiBs, particularly in electric vehicle applications [53]. Lithium insertion/de-insertion systems rely on their particle dimension, crystallinity, form, and surface region. TiO_2_ has many allotropic formations, including rutile, anatase, and brookite. Anatase has been suggested as the best electroactive structure; the remaining two (brookite and rutile) have been thoroughly researched for application in negative electrodes. By combining these two structures, hydrothermal reaction (temperature at 150 °C) and annealing operation (temperature at 300 °C) can be used to create TiO_2_ nanotubes [23,61,62]. Materials with nanostructured anodes, such as porous TiO_2_-C nanocomposite carapaces, TiO_2_–graphene nanocomposite, as well as graphene-assisted anatase TiO_2_, demonstrated over 90% capacity retaining as well as greater than 100 cycle steadiness [63,64,65]. However, the primary obstacle to the widespread commercialization of TiO_2_ as an anode material is that it has poor thermoelectric capability (175 mA/h g). This is especially true when combined with cathode materials of higher capacity [62].

##### Conversion

This section gives an overview of conversion metal compounds and oxides employed in LiB anodes. An electrochemical reaction mechanism combining these chemical compounds and lithium implies the decline (oxidization) of the conversion metal and the composition of lithium compounds. Such compounds with anode bases provide excellent reversible capacities, ranging from 500 to 1000 mAh/g, where numerous electrons are joined to the transition [66,67,68].

i. Oxides of iron

For LiB anode materials, nanostructured iron oxides are essential. For example, oxides of iron (α-Fe_2_O_3_, γ-Fe_2_O_3_, and Fe_3_O_4_) are better than carbon/graphite and semiconductor materials as anode materials. Such iron oxides have properties like reactivity with lithium conversion, high theoretical capacities (about 1000 mAh/g), non-toxicity, and low cost, which makes them fitted for anode applications [62,69,70,71]. However, they have limitations such as low cycle life due to little ionic conductivity (10^−7^ S/gm at RT), limited Li-ion diffusion, volume change, and iron accumulation in the cycling process [72]. To tackle these limitations, many different nanostructured iron oxide preparation methods with various sizes, shapes, and porosity, such as 1D nanorods [73], 2D nano-flakes [74], 3D architecture with high porosity [75], Fe_3_O_4_ nanocomposites [76], Fe_3_O_4_ nanotube arrays [77], and so on, have been improved with nanoengineering to retain high capacity and stability [78,79,80,81].

Yin et al. [82] studied α-Fe_2_O_3_ nanofibers, which were synthesized by organic compounds through the annealing operation (at 500 °C) in the inert environment for 3 h. The organic compounds become decomposed as a result. Further heat treatment (at 500 °C) is given to obtain a phase transformation into γ-Fe_2_O_3_ from α-Fe_2_O_3_ in the argon environment for 6 h. In another study, Wu et al. [83] fabricated α-Fe_2_O_3_ nanorods while studying the size and morphology.

ii. Cobalt oxides

Cobalt oxides (CoO and Co_3_O_4_) are used as negative electrodes for LiBs because they have some outstanding redox properties, such as high theoretical capacity (Co_3_O_4_ is 890 mAh/g while CoO is 715 mAh/g) [84], capacity retention, and so on. However, it has some limitations, such as toxicity, volume change, and semi-conducting nature during charging and discharging, leading to the hindering of mass practical applications [62,85]. Like other materials, it comes in different forms like nanowires, nanosheets, porous nanostructures (nanoplates), multiple shells–spheres, Co_3_O_4_ nanotubes. These materials are synthesized for anode applications to address the above issues [86,87,88].

Guan et al. [89] fabricated CoO octahedral nanocages in a pure phase. The authors employed NH_3_ (agent for coordination etched) through template-free methods. Hence, uniformly sized octahedral nanocages with edge lengths between 100 and 200 nm were produced. Finally, the anode materials have outstanding cycle performance with a better rate capacity due to enhanced lithium storage capacity. More importantly, those nanocages provided higher specific capacity and current (474 mAh/g at 5C). Their high capacity and high-rate capabilities are achievable due to the enormous voids that exist and the handling of significant volume fluctuations.

##### Alloys

Alloys play an integral role in developing specific capacity, which is an elemental parameter that is considered in novel anode material for future LiBs. It is thought that LiBs can meet the power demands of portable electronics, HEVs, and electric cars. Some materials such as germanium (Ge), silicon (Si), tin oxide (SnO_2_), silicon monoxide (SiO), etc. respond to lithium regarding the process of alloying and de-alloying. SnO_2_ has 783 mAh/g (theoretical specific capacity), but Si has 4211 mAh/g (theoretical specific capacity) [90,91,92].

i. Silicon

Silicon (Si) is better than graphite as an active material for anode because of its ubiquity and favorability to the environment. Si reduces donor–acceptor interaction duration and increases the performance rate because of its high volumetric capacity (9786 mAh/cm^3^) [23,65,93,94,95,96]. It is clear why Si as well as its subsidiary products are regarded as the most promising substance for the creation of future LiBs. This accounts for significant interest in both the academic and industry communities in the development of materials’ potential as anode active materials. Extensive research has been conducted on Si negative electrodes, particularly electrochemical lithiation. The high specific capacity value has been explained as resulting from the synthesis of intermetallic Li-Si binary composites, including Li_7_Si_3_, Li_12_Si_7_, Li_13_Si_4_, and Li_22_Si_5_ [97].

In contrast, a major challenge preventing the extensive adoption of silicon-based anodes is the large volume changes that occur throughout the lithiation/de-lithiation process, which produces poor cycling performance. To tackle this issue, researchers are currently employing novel materials such as nano additives. Several allotropes of silicon nanomaterials, such as silicon nanotubes, nanowires, and sponges, can be employed as LiB anodes. Some techniques such as molten salt, electrodeposition, chemical vapor deposition (CVD), and so on are used to create these structures [94,98,99].

In another study, Chan et al. [22] use Si nanowires as an anode, and they demonstrate notable electrochemical performance when compared to various forms of Si. Excellent benefits resulted from the silicon nanowires being grown directly on the stainless current collector. Compared to bulk silicon, nanoscale silicon materials can withstand greater volume changes without breaking. Si nanowires were electrically coupled to the current collector, allowing for quick charge transfer and complete silicon utilization.

ii. Germanium

Germanium-based anodes are necessary for lithium-ion batteries compared to graphite anodes because they offer extremely more electrical conductivity—10,000 times greater compared to Si and have little energy/band gap (0.67 eV), high theoretical capacity (1623 mAh/g), and high Li-ion diffusion [62,92,100]. Li-ions migrate into Germanium (Ge) 15 times faster than Si at room temperature as well as 400 times faster at 360 °C. Thus, the efficient charge transport and rate performance of Ge is more than Si [101]. Hence, Ge is vital in high-power density usages in EVs of high-power capability. However, when it comes to bulk structure, Ge is limited in practical applications because of high volume change (about 300%) caused by mechanical stress during insertion/de-insertion [102]. To tackle this issue, nano-forms such as nanoparticles [103], nanowires [101], and nanotubes [104], and so on, can effectively suppress the volume changes with high efficiency compared to bulk materials and micro structured materials, leading to a flexible form at elevated temperature.

When it comes to nanostructured germanium Yuan et al. [105] investigated the alkanethiol-passivated Ge nanowires (NWs) for LiB anodes. They first synthesized the Ge NWs with a gold-seeded growth process. Next, a hydrofluoric acid solution was applied to create hydrogen-terminated Ge NWs, which were then surface-passivated with thiol groups. The thiol-passivated Ge NWs showed exceptional electrochemical performance, including better reversible specific capacity of 1130 mAh·g^−1^ and robust cycling stability at 0.1C. In addition, these NWs were shown to have high-power capabilities, maintaining a reversible capacity of 550 mAh·g^−1^ even at an elevated charge of 11C. To further elucidate the effect of passivation on germanium nanowires in lithium-ion batteries, comparative experiments were also performed using Ge nano-substances and un-passivated Ge NWs. Table 2 summarizes the electrochemical properties of nanomaterials used in the anodes of lithium-ion batteries.

#### 3.1.2. Positive Electrode

**Figure 9:**
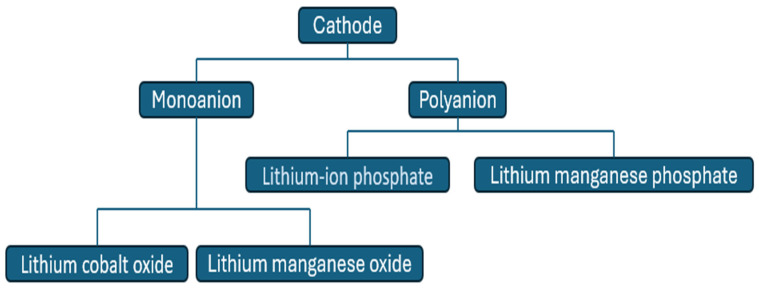
Types of cathode materials and their subcategories.

The cathode is important for LiBs in terms of facilitating the reduction reaction. Highly porous forms are thus essential for Li-ion diffusion. Nanostructured materials play a vital role in achieving these characteristics [106,107]. Figure 9 illustrates the various classes of cathode materials and their subclasses.

##### Monoanion

i. Lithium cobalt oxide (LCO)

Lithium cobalt oxide (LCO) has been the most widely used positive electrode substance for LiB since 1991, when it was first commercialized [108,109]. Its advantages include low self-discharge, enhanced capacity (274 mAh/g) [110], and longevity. However, cost, poor rate performance, and less thermal stability because of the exothermic reaction on the cathode side make it less desirable in certain applications [108,111]. To tackle these issues, the nanosized LCO approach is adopted because it enhances the surface region and reduces Li-ion migration distance, leading to the development of electrochemical kinetics for LCO [112]. Some techniques, such as the hydrothermal process [113], post-templating [114], and spray drying [115], are used to synthesize LCO.

The critical size of high-temperature nanostructured materials has been demonstrated to be approximately 15 nm, particularly HT-LCO (high-temperature lithium cobalt oxide) nano-substances [116]. When the nanosubstance falls less than 15 nm, the HT-LCO’s specific capacity decreases because Co^3+^ is reduced to Co^2+^. To achieve great performance without a significant capacity loss, it is preferable to restrict at least one dimension to lie between 15 and 30 nm for HT-LCO nano-substances while reducing capacity deduction [117].

ii. Lithium manganese oxide (LiMn_2_O_4_)

Lithium manganese oxide (LMO) is a promising material with a spinel crystalline form compared to common positive electrodes. This is because it is less expensive, less toxic, and has similar traits to LCO [118]. However, some issues like low diffusion of Li-ions, transfer rate, less stability at high temperature, and capacity loss need to be addressed to achieve high performance in LiBs [119,120,121]. When it comes to the reduction in the Li-ion migration distance, the use of nanowires and nano substances have been proposed by some studies [122,123,124].

Labyedh et al. [125] introduced a groundbreaking synthesis technique by sequentially applying layers of electrolytic manganese dioxide and Li_2_CO_3_ onto a flat substrate at 3500 °C, utilizing a pillar array. They observed the aspect ratio surpassing 20. This was followed by the solid-state reaction that deposited a uniform and crack-free film of spinel LiMn_2_O_4_. Therefore, a capacity of 0.4 mAh/cm^3^ (compared to theoretical capacity: 1.27 mAg/cm^3^) was reached for these 3D electrodes when operated at an extreme temperature, i.e., 100C (1C = 17.8 μA/cm^2^).

##### Polyanion

Polyanion means more than one anion compound in the cathode composition. Hence, such a compound structure is more complicated and functionalized.

***Olivine materials***

LiMPO_4_ is an olivine material that is employed as a cathode for high-power sources like EVs. Its capability can be improved by decreasing the particle size of active materials electrochemically. Hence, small ionic and electronic conductivity are effective for high performance [126]. One of the more appealing cathode materials is LiFePO_4_ (LFP), which was originally synthesized by Goodenough in 1997 [87]. Its thermal stability results in a remarkable safety status, low toxicity, and an appropriate gravimetric capacity (170 mAh/g) [127]. The substance’s ionic and electrical conductivity, however, still has potential for development. The poor electrochemical performance of LFP at low temperatures is one of the primary difficulties that need to be considered [128].

It has been demonstrated that altering the size and shape of particles is an efficient way to address these problems. Peng et al. [129] produced LiFePO_4_ nanowires with self-assembled single crystals covered in a consistent 2 to 3 nm thick uniform coating of amorphous carbon. The LFP material was created at 200 °C via a solvothermal reaction that used tetra-ethylene glycol as well as the inclusion of FeCl_2_.4H_2_O, ascorbic acid, LiOH, H_2_O, and LiH_2_PO_4_. Many 40-nanometer-diameter self-assembled nanowires made up the final structure. When the produced LFP material and industrial LiFePO_4_ particles were compared, the former’s lithium-ion diffusion coefficient was higher (1.82 × 10^−14^ cm^2^/s against 5.62 × 10^−15^ cm^2^/s). Three factors account for this conductivity: a greater penetration of electrolytes through positive electrodes, the presence of many active sites, and a shorter diffusion route length for lithium ions. When compared to industrial LiFePO_4_-based devices, the synthesized LFP also demonstrated better storage performance, with specific capacities of 150 (1C) and 110 mA h/g (30C, 1C = 0.17 A/g) as opposed to 130 (1 C) and 60 mAh/g (30C, 1C = 0.17 A/g).

**Figure 10:**
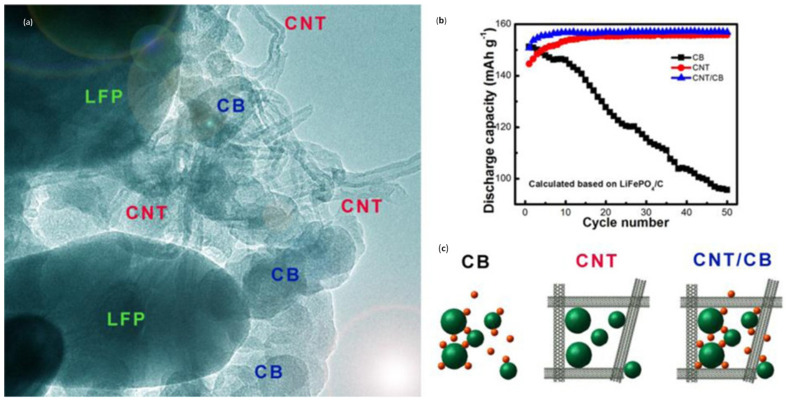
(**a**) Modification of the LFP cathode with CNT or CB (TEM images), (**b**) cycle number vs. capacity relation with CB, CNT, and CNT/CB. (**c**) Schematic of CB (red spheres), CNT (gray tubules), and CB/CNT network as a conductive additive for LiFePO_4_/C (green spheres) composite cathodes. [131]. © ACS, 2014.

It has also been suggested that Klason Lignin could be used as a cathode-active electrode [130]. When a three-dimensional conducting structure incorporates graphene and/or CNTs into the cathode, the spherical particles are taken by a granular cathode. Li-ion transport is quick for nanostructured cathodes due to limited diffusion paths, which is crucial for batteries that function at high current densities. Using hierarchical carbon nanotube/carbon black (CNT/CB) scaffolds, Liu et al. [131] created a 3D conducting structure for the LiFePO_4_ cathode (see Figure 10). This scaffold cathode shows higher performance and electrochemical kinetics due to shorter Li-ion dispersion length. A functioning cathode’s network of interlinked carbon nanotubes (CNTs), graphene, carbon black, and carbon layers functions as three-dimensional electron paths. The pores that are related to one another can be used as channels for Li-ionic dispersion.

#### 3.1.3. Electrolyte

The electrolyte is a crucial element in LiBs, as it facilitates Li-ion’s transportation between positive and negative electrodes. LiB electrolytes are generally categorized into two main types: liquid electrolytes (LEs) and solid polymer electrolytes (SPEs). While solid polymer electrolytes (SPEs) offer distinct benefits, they also face challenges, such as physico-chemical opposition for negative electrodes, which can hinder ion conduction. For this reason, LEs remain the preferred choice in lithium-ion battery (LiB) applications. Advancing electrolyte technology, particularly with nanomaterials, is vital for enhancing the capacity and security of LiBs [132]. The idea that nanoparticles could improve the characteristics of the traditional liquid electrolytes used in rechargeable lithium batteries may seem unexpected at first, but there is now solid proof that they can. The conductivity of non-aqueous electrolytes can be increased by a factor of six by adding powders, particularly in nanoparticulate form of substances like Al_2_O_3_, SiO_2_, and ZrO_2_ [133].

**Figure 11:**
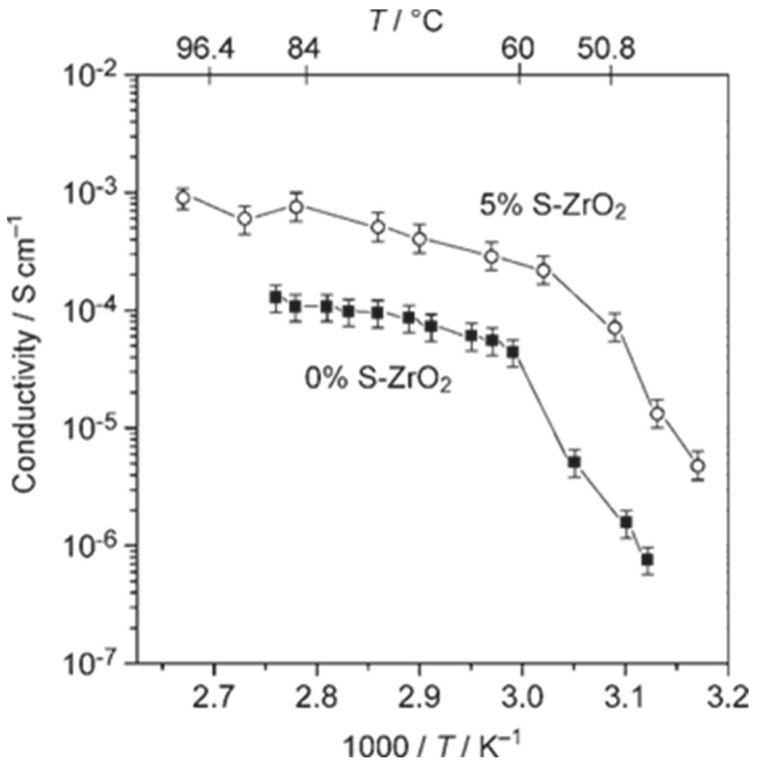
Relation between conductivity and temperature with 5% and 0% of S-ZrO_2_, two containing the PEO_8_LiBF_4_ mixing [139]. © Annals of the New York Academy Science, 2006.

To advance lithium batteries, it is important to note that the creation of polymer electrolytes that conduct only cations and are solvent-free is seen as crucial. There have been reports of attempts, mostly aimed at immobilizing the anion in the polymer framework, but with only limited success because this method typically lowers the total conductivity of the electrolyte [134]. Figure 11 illustrates this improvement by contrasting the Arrhenius plots of an electrolyte with S-ZrO_2_ filler and the identical electrolyte without filler. It is evident that throughout the whole temperature range, the conductivity of the electrolyte containing S-ZrO_2_ is higher than without S-ZrO_2_ [135].

Another nanostructured substance utilized as an electrolyte and ion transport component in LiBs is methacrylate functionalized SiO_2_ (MA-SiO_2_). These mesoporous nano-substances include inner-pore narrows that offer the ideal route for Li-ions to go through the inner lattice of the material, according to Shin et al. [136]. After thorough research, it was shown that the use of interconnected combined gel polymer electrolytes containing methacrylate-functionalized mesoporous nano-substances of silicon dioxide (SiO_2_) can significantly improve the ability to cycle lithium-ion battery (LiB) cells, especially at elevated temperatures of operation.

LLZTO (lithium lanthanum tantalum zirconate), a ceramic compound, is a crucial material that enhances lithium conduction. Its rigid structure not only promotes ionic transport but also offers a robust framework that mitigates the growth of lithium dendrites, a common issue in lithium batteries leading to short circuits and reduced battery life [137]. Additionally, LLZTO is renowned for its high ionic conductivity, a crucial characteristic for improving the overall performance of solid-state batteries. This property facilitates efficient lithium-ion transport within the electrolyte matrix [138]. Additionally, LLZTO is renowned for its high ionic conductivity, a crucial characteristic for improving the overall performance of solid-state batteries. This property facilitates efficient lithium-ion transport within the electrolyte matrix.

Chen et al. [137] identified PEO as another crucial component that serves as a polymer matrix in composite electrolytes. The authors determined that PEO contributes to the mechanical flexibility and viscoelasticity of the electrolyte, which is essential for accommodating the volume changes during battery charge and discharge cycles. This flexibility is critical for maintaining the integrity and performance of the battery over time. The incorporation of LiTFSI (lithium bis(trifluoromethanesulfonyl)imide), in addition to LLZTO and PEO, creates a composite electrolyte that not only enhances the electrochemical performance of lithium batteries but also addresses challenges related to mechanical stability and dendrite formation [137].

Furthermore, Sun et al. [138] employed ceramic–polymer composite electrolytes that incorporate inorganic fillers, such as LLZTO, into polymer matrices. This strategy enabled them to achieve both high ionic conductivity and mechanical flexibility. The researchers found that the inclusion of LLZTO in the composite polymer electrolytes significantly enhanced ionic conductivity, particularly at low mass contents below the percolation threshold. This improvement in ionic conductivity resulted in better lithium-ion transport within the electrolyte. Overall, the CPEs maintained chemical and electrochemical stability while providing the necessary mechanical flexibility to accommodate volume changes during battery operation, thereby enhancing the overall safety and longevity of the batteries [137,138].

#### 3.1.4. Separator

Separator is vital for developing the capacity of LiBs. It acts as an electrical insulator between electrodes but allows ions to flow between electrodes. Separators need to demonstrate strong machine-like steadiness as well as chemical resilience for avoiding decay at the interfaces among the positive electrode, electrolyte, and negative electrode, particularly stimulated by the presence of contaminations [140]. Generally, there are three types of separators: polypropylene (PP), polyethene (PE), and their mixtures. However, its increased concern for low-temperature (PP at 160 °C and PE at 130 °C) melting leads to fire, short circuits, and overheating.

In addition, commercial LiB separators present drawbacks such as melting–shortening, dendritic emergence while charging and discharging cycles, as well as difficulties with wetting tendency. These factors hinder electrolyte performance, which in turn degrades the battery’s overall efficiency [141,142]. Several techniques, such as forming polymer–ceramic nanocomposites, applying nanoceramic coatings onto polymers, and using nanoceramics directly, are employed to integrate nanomaterials into LiB separators. Coating is particularly preferred compared to other techniques because of ease of use [143]. Composite separators with enhanced characteristics are created when inorganic particles are added to porous or nonwoven separators. Specifically, SiO_2_, Al_2_O_3_, and TiO_2_ (ceramic elements) are added to polymer hosts to improve their durability, wetting, electrical conductivity, and thermal endurance [144]. Inorganic particles can be integrated into microporous membrane separators by coating them with a polymer binder, resulting in ceramic-coated composite membranes. Alternatively, these particles can be embedded in a polymer matrix, forming ceramic-filled combined membranes.

In composite membranes featuring ceramic particle coatings, powders that are sold commercially like Al_2_O_3_, SiO_2_, and TiO_2_ are dispersed in a polymer solution. This mixture is then applied to microporous polypropylene (PP) or polyethylene (PE) (membrane separators). The polymer serves as a partnership, securing non-organic atoms to separators [145,146]. On the front side of a permeable polyethylene (PE) separator for instance, core–shell patterned silica-poly (methyl methacrylate) (SiO_2_-PMMA) sub-microspheres were applied to create an effective ceramic-coated separator [147]. PMMA shell enhances absorption and retention of liquid electrolytes. This innovative separator exhibits reduced thermal shrinkage, improved wettability with liquid electrolytes, and exceptional electrolyte retention capabilities.

**Figure 12:**
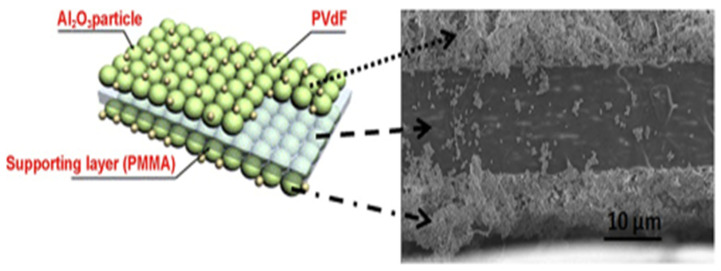
Separator with inorganic and organic tri-layer through the schematic diagram and SEM image [148]. © Elsevier, 2010.

Additionally, the addition of ceramic particles improved cycle performance and C-rate capabilities. Inorganic particle layers have also been dip-coated on two surfaces of a PMMA thin film to create tri-layer composite membranes [148]. To create the coating mixture, Al_2_O_3_ and a PVdF-co-HFP binder were mixed in a 9:1 filler-to-binder weight ratio (see Figure 12).

Boron nitride nanotubes (BNNTs) have been utilized to improve the insulating characteristics of separators in LiBs, effectively preventing short circuits. These nanomaterials offer more thermal conduction, an excellent wetting tendency for liquid electrolytes, as well as efficient ion transport with electrodes as well as electrolytes. Boron nitride nanotubes (BNNTs) are considered promising for applications requiring high-rate and high-temperature performance, like in electric vehicles (EVs) as well as the power-grid process. Their exceptional thermal stability and mechanical strength make them suitable for these demanding conditions [149].

### 3.2. Nanotechnology Application in Sodium–Sulfur Batteries

**Figure 13:**
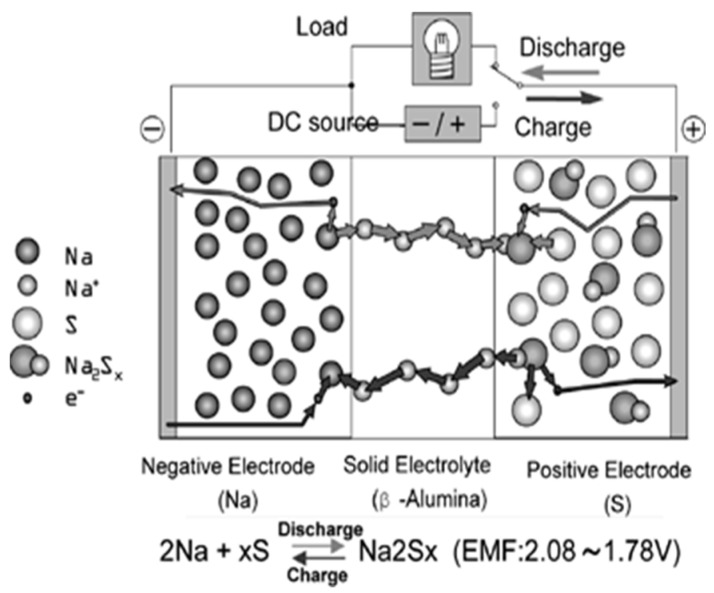
Operating principle of sodium–sulfur battery cell [150]. © John Wiley and Sons, 2005.

Sodium–sulfur (Na-S) batteries are secondary batteries using sodium and sulfur for the negative and positive electrode components, respectively. A sodium-ion-conductive beta-alumina ceramic as the electrolyte [150]. Operating at around 300 °C, these batteries keep their electrode materials molten, reducing resistance for sodium ion flow through the solid electrolyte. They perform a reversible electrochemical reaction during charging and discharging. The nominal EMF of a Na-S cell is about 2 V. Though relatively new, Na-S batteries provide superior energy density, higher durability, and low environmental impact; thus, they are well suited for extensive usage in electric vehicles, grid energy storage, and backup power. Figure 13 provides an illustration of the operating principle of the battery cell of a typical Na-S battery.

#### 3.2.1. Electrode Materials

Zhao et al. [151] highlight the significant application of nanomaterials and their technology in enhancing the performance of materials used for electrodes of sodium–sulfur batteries under normal temperature. Regarding sodium metal anodes, this study emphasizes the use of nanostructured host materials to mitigate the problems that come with the dendrite growth of sodium metal and volume expansion during cycling [152]. These host materials, often carbon-based or incorporating metal compounds, provide a porous and conductive framework for Na deposition, promoting uniform plating and preventing dendrite formation [153,154,155]. The incorporation of transition metal nanoparticles or single atoms within these nanostructured hosts further enhances their sodiophilicity (how well a material interacts with sodium) and improves the stability of the interphase of the solid electrolyte [156,157,158].

For the cathode side, nanotechnology is vital in addressing the challenges of sluggish redox kinetics, volume expansion, and the polysulfide shuttle effect [159,160]. The authors discuss the use of nanocomposite catalytic cathodes, where various nanomaterials, such as metal oxides, sulfides, and single atoms, are incorporated into porous carbon hosts to accelerate the conversion of sulfur species and enhance reaction kinetics [161,162]. The authors highlight the advantages of single-atom catalysts, which offer maximum catalytic utilization and high activity due to their homogenous distribution and unique atomic configurations [163,164].

Further studies by Tang et al. [165] investigate the use of vanadium carbide nanoparticles, which have been ingrained in carbon nanofibers (VC-CNFs), as a 3D self-supported cathode for sodium–sulfur batteries at normal temperature. The electrochemical performance of this specific cathode was evaluated at room temperature, and its interaction with polysulfides was investigated using adsorption experiments, XPS, UV–Vis absorption, and symmetric cell CV and EIS measurements. The VC-CNFs cathode was found to exhibit a better performance in its electrochemical properties with a capacity retention of 96.2%. This favorable outcome was due to the harmony of the “confining—trapping—catalyzing” mechanism provided by the VC-CNFs structure [165]. Also, like other studies, the vanadium carbide nanoparticles acted as chemical trappers, adsorbing sulfur species and mitigating the shuttle effect while also serving as electrocatalysts, accelerating the redox reactions of polysulfides, and improving the reaction kinetics. This finding concurs with that from a previous study by Cai et al. [166]. Tang et al. [165], however, focused on liquid-state Na-S batteries and did not consider the effect of VC-CNFs cathodes in solid-state sodium–sulfur batteries with advanced solid electrolytes.

#### 3.2.2. Electrolyte and Separator

A review of relevant publications shows a dearth of knowledge regarding the use of nanotechnology in electrolyte materials as well as separators in sodium–sulfur batteries. In a similar study, however, Ma et al. [167] explore the usage of solid-state electrolytes in Na-S batteries operating at room temperature to enhance safety and prevent issues like polysulfide shuttling and dendrite formation. The authors employ an FSA-Na solid-state electrolyte membrane as both the electrolyte and separator in their battery design, which uses a perfluorinated sulfonic resin powder in the form of sodium. This study highlights the advantages of this solid-state electrolyte in controlling the shuttle effect and making the battery more stable [168,169]. Although they do not explicitly mention the use of nanotechnology in the electrolyte itself, the utilization of a solid-state electrolyte membrane represents a departure from traditional liquid electrolytes and aligns with the broader trend of employing advanced materials and nanotechnology in the development of next-generation batteries.

Despite the progress made thus far, Na-S batteries still face limitations in terms of their ability to retain enough capacity as well as a prolonged lifespan, primarily due to the inherent challenges associated with sodium’s reactivity and the formation of polysulfides or discharge products [170,171,172]. To overcome the drawbacks of using Na-S batteries, Sahgong et al. [173] suggest exploring novel sodium-ion conducting separators, developing new catalysts to suppress dendrite formation, and investigating innovative materials and designs to enhance battery performance. Table 3 provides a summary of the most common applications of nanomaterials in Na-S batteries.

### 3.3. Nanotechnology Application in Redox Flow Batteries

**Figure 14:**
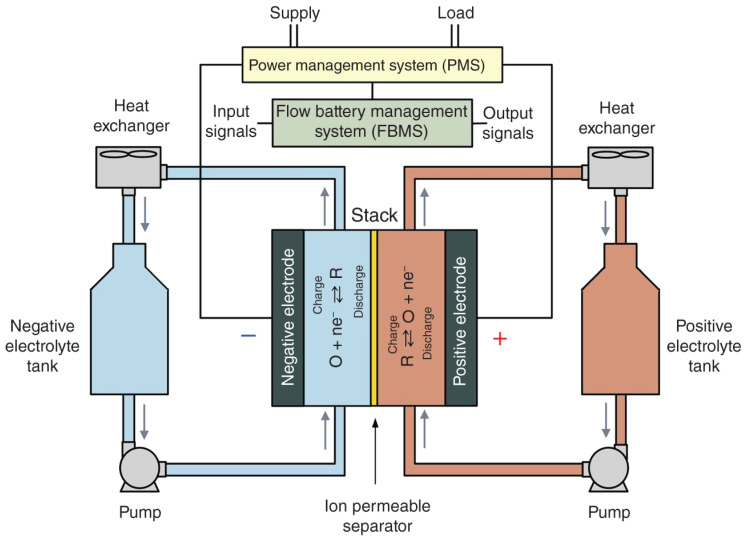
A flow battery cell with its main components [182]. © John Wiley and Sons, 2024.

Redox flow batteries (RFBs) primarily store energy that operates by converting electrical energy into chemical energy and back again [174,175,176,177]. Unlike regular batteries that store energy inside their electrodes, RFBs use special liquids called electrolytes to store energy. This gives them advantages like flexibility, long life, and safety [175,178,179,180]. RFBs are great for storage solutions that require enhanced capacities, such as keeping the power grid stable, improving power quality, and helping to use renewable energy sources [174,181]. They have two tanks with electroactive electrolytes, two electrodes, a separator, and a system to circulate the liquids [174]. When charging, the liquids undergo chemical reactions at the electrodes to store energy. When discharging, the reverse happens, releasing the stored energy as electricity. RFBs can be adjusted to fit different energy needs because their power and energy capacity can be scaled independently [174]. Figure 14 shows the components of the cell of a flow battery as well as its working principle.

#### 3.3.1. Electrode Materials

Nanotechnology has emerged as very influential for enhancing the performance of electrodes in the redox flow battery (RFB). The integration of nanomaterials into carbon-based electrodes has been shown to tackle major deficiencies like low conductivity, sluggish reaction kinetics, and high costs [183]. Metal nanoparticles like platinum, palladium, gold, and iridium have been incorporated to improve the conductivity and electrocatalytic activity of electrodes [184,185]. However, the high cost and potential for side reactions, such as hydrogen evolution, associated with noble metals have led to the exploration of non-noble metal alternatives like bismuth, copper, and tin [186,187,188,189]. These non-noble metals not only offer cost advantages but also mitigate undesirable side reactions, contributing to improved efficiency and durability.

Metal oxide nanoparticles have gained significant attention as a replacement for precious metal catalysts because they are more economical and have a high catalytic activity [185,186,187]. These nanoparticles, including CeO_2_, MnO_2_, ZrO_2_, Ta_2_O_5_, and NiCoO_2_, have been shown to enhance reaction kinetics and reversibility, particularly at the positive electrode where reactions involving water molecules often limit overall cell performance [188,189,190,191,192]. Furthermore, some metal oxides, such as SnO_2_, Nd_2_O_3_, and NiMn_2_O_4_, exhibit bifunctional catalytic behavior, promoting both positive and negative redox reactions [193,194,195,196]. In addition to metal and metal oxide nanoparticles, metal sulfides like MoS_2_ and CoS_2_/CoS heterojunctions have been investigated for their ability to facilitate redox reactions, especially in polysulfide-based RFBs [197,198,199]. These materials have demonstrated promising results in improving the electrochemical kinetics and mass transport of polysulfide couples.

Carbon-based nanomaterials have also been widely explored because of their intrinsic features like greater surface area, superior conductivity, and chemical stability [200,201,202,203,204,205]. These materials include carbon nanofibers and carbon nanotubes. The integration of these nanomaterials into electrodes has been shown to enhance mass transport, charge transfer, and electrocatalytic activity, leading to improved cell performance. Carbon-based nanomaterials, particularly the graphene-based ones like graphene and graphene oxide, have garnered significant attention [206,207,208,209,210,211]. Their large surface area, exceptional conductivity, and stability in acidic environments ensure they are attractive candidates for electrode modification. Research has shown that these materials can improve electrocatalytic activity, reduce side reactions, and enhance the durability and overall performance of flow batteries [212,213].

Overall, the application of nanotechnology in electrode materials represents a dynamic and promising research area related to flow batteries. Altering the characteristics of electrodes through the incorporation of nanomaterials offers a pathway to overcome existing limitations and achieve high-performance, cost-effective, and durable energy storage solutions. Continued exploration and development in this field are expected to drive notable progress in the performance and commercial viability of RFB technologies.

#### 3.3.2. Electrolyte Materials

The incorporation of nanotechnology into the electrolytes of redox flow batteries (RFBs) has garnered significant attention to enhance their performance. Researchers have explored the use of nanoparticles to create suspended nanofluids, which exhibit intermediate properties between liquids and solids, aiming to improve the electrochemical reaction kinetics, electron transport, and ion transport within the battery. Early attempts to utilize micron-scale particles as conductive additives in electrolytes showed promising results in terms of increased electrochemical activity [214]. However, the accompanying increase in viscosity led to higher pump power losses, negatively affecting the efficacy and durability of the battery. To address this challenge, subsequent research has shifted focus towards nanoscale particles, offering the potential for improved performance without the drawbacks associated with larger particles.

Metal-based nanoparticles have been investigated for their ability to enhance electron migration within the electrolyte due to the rapid propagation of electrons within these particles [215]. However, challenges such as increased viscosity and potential instability have prompted further exploration of alternative nanomaterials. Carbon-based nanofluids, particularly those incorporating graphene and carbon nanotubes, have emerged as promising candidates because of their inherent features such as large surface area, porous configuration, and very good conductivity [216,217,218]. These nanofluids have demonstrated the ability to enhance the conductivity and electrochemical reaction kinetics of the electrolyte, contributing to improved battery performance.

Extensive research is necessary to sufficiently appreciate the mechanisms underlying the impacts of nanoparticles on electrolyte properties and battery performance. The type, size, concentration, and surface morphology of nanoparticles can all influence the physical and electrochemical characteristics of the electrolyte, and careful optimization is required to realize the necessary trade-off between viscosity and active species utilization [219]. Employing computational modeling and simulation will be key in elucidating the complex interactions between nanoparticles and electrolytes, enabling the creation of more effective nanofluid electrolytes for RFBs [220,221,222,223,224,225,226]. Additionally, advanced characterization techniques are needed to probe the ion and electron transport pathways and exchange mechanisms within these systems, providing deeper insights into their behavior and facilitating further optimization.

#### 3.3.3. Separator Materials

The application of nanotechnology to enhance separator materials in redox flow batteries (RFBs) primarily focuses on addressing the limitations of conventional ion-exchange membranes, such as Nafion. While Nafion boasts high ionic conductivity and chemical stability, its permeability to active species can contribute to crossover and diminish the performance of the battery [227,228,229]. To mitigate this problem, researchers have explored the incorporation of diverse organic and inorganic nanomaterials into Nafion membranes, aiming to improve selectivity and reduce crossover [230,231,232]. For instance, the addition of polymers like poly (4-vinyl pyridine), polypyrroles, and polyaniline, or inorganic nanoparticles like silicate (SiO_2_) and zirconium phosphates (ZrP), could modify the Nafion matrix, creating a more tortuous path for ion transport and hindering the passage of larger active species.

Another strategy involves the fabrication of composite membranes that integrate Nafion with other cost-effective, high-performance materials [233,234]. Sulfonated poly(ether ether ketone), or SPEEK for short, known for its low ion permeability and chemical stability, has been employed as a substrate material for Nafion. The resulting Nafion-SPEEK composite membranes demonstrate enhanced selectivity and reduced crossover while preserving favorable ionic conductivity. Furthermore, anion exchange membranes have been explored as alternatives to cation exchange membranes like Nafion [235,236,237,238]. These membranes, typically synthesized through chloromethylation of a polymer substrate followed by quaternization of an amination reagent, leverage electrostatic repulsion between their cationic groups and the positively charged active species to further curtail crossover. Recent progress has also witnessed the utilization of polyacrylonitrile nanofiltration membranes in vanadium redox flow batteries (VRFBs) [176,239].

These membranes employ pore size exclusion to achieve high selectivity, presenting a novel design concept that broadens the material choices for separators beyond conventional ion exchange resins. In essence, the integration of nanotechnology into separator materials for RFBs remains a vibrant research field, with ongoing endeavors to develop innovative nanocomposite membranes exhibiting superior selectivity, conductivity, and stability. The overarching objective is to realize high-performance, cost-effective, and durable separator materials that can substantially elevate the efficiency, capacity, and lifespan of RFBs, thereby promoting their widespread implementation for more extensive use in energy storage systems. A summary of nanomaterial application in flow batteries is presented in Table 4.

### 3.4. Nanotechnology Application in Supercapacitors

**Figure 15:**
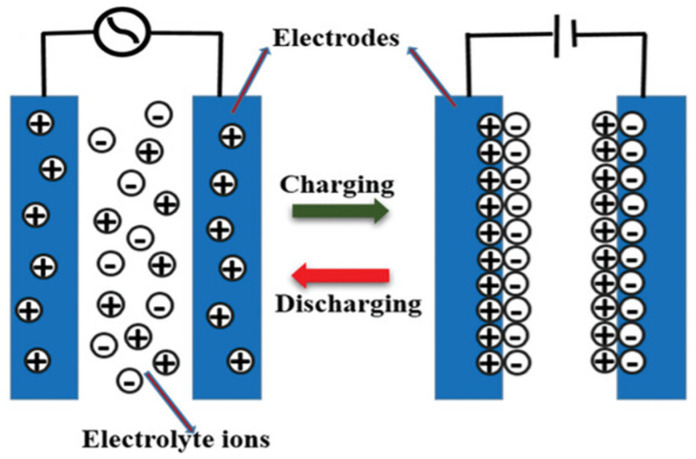
Operating principle of an electrochemical double-layer capacitor [242]. © John Wiley and Sons, 2023.

Supercapacitors (SCs) are devices used to store energy, similar to batteries but with different energy storage mechanisms [240]. They offer several advantages over batteries, such as fast charging, reliability, long cycle life (over 100,000 cycles), absence of toxic metals, wide operating temperatures, and the ability to deliver more power than batteries and store more energy than traditional capacitors. These properties position supercapacitors as a favorable alternative for extensive application in energy storage solutions in electronics, motor vehicles, and industrial machinery [241]. The mode of operation of an electrochemical double-layer capacitor is illustrated in Figure 15. Several studies have been undertaken to improve the properties of supercapacitors; those that involve nanotechnology are highlighted here.

To improve the running of SCs, Boyea et al. [241] investigate the potential of carbon nanotubes (CNTs) as electrode materials. The researchers fabricated electrochemical capacitors with CNT electrodes, then compared these with traditional activated carbon electrodes. They analyzed how nanotube growth and processing, including purification and functionalization, affected performance. Initial results suggest that CNT-based supercapacitors could significantly boost power and energy density with further development. CNTs offer several advantages, such as greater conductivity, larger surface area, and improved corrosion resistance, over activated carbon [241]. Optimizing the electrolyte composition and electrode material is crucial for optimal performance.

Nanotechnology has significantly boosted supercapacitor electrodes’ energy storage by customizing electrode materials’ nanoscale size, shape, and arrangement. Tailored nanostructures create electrodes with hierarchical pores, improving ion access and charge storage [243,244]. Advanced carbon nanostructured materials like carbon nitride, carbon quantum dots, and doped graphene further enhance electrodes’ surface area, electrical conductivity, and the improved capacitance [245,246,247]. Employing 2D nanomaterials like transition metal dichalcogenides, graphene, and boron nitride has led to electrodes with an enhanced surface area, improved conductivity, as well as unique quantum confinement effects [248,249]. Furthermore, binder-free electrodes overcome traditional binder limitations, resulting in higher active material loading, better ion diffusion kinetics, and enhanced electrochemical stability.

Recent studies have explored the use of metal–organic frameworks (MOFs) and covalent organic frameworks (COFs) in electrode materials of supercapacitors and rechargeable batteries. MOFs and COFs have a customizable crystalline structure in addition to permanent porosity, which sets them apart from more conventional porous substances like zeolite or active carbon. As a result, these crystalline porous materials (CPMs) could have more accurate atomic-level design and operation [250,251]. These benefits enable us to address the electrochemical energy storage (EES) problems. MOFs and COFs have been thoroughly investigated up to this point and show great promise in EES systems in several ways, including direct use as active materials [252] and as templates and precursors to produce derived porous carbon/metal oxides/metal sulfide [253].

Li et al. [254] initially employed MOF177 as the anode material, and Férey et al. [255] used MIL-53 as the cathode material. CPMs have therefore been widely studied as electrode materials for LiBs and supercapacitors. These studies have served as inspiration for CPMs, which are regarded as desirable candidates in rechargeable batteries due to their affordability, excellent stability, porosity, and ease of fabrication. Additionally, conductive substances in supercapacitors have undergone extensive development. For instance, the ZIF67-polypyrrole network was created in a study by Xu et al. [256] for a flexible supercapacitor. A large areal capacitance of 225.8 mFcm^−2^ was attained by the electrode.

Finally, by combining multiple nanomaterials, nanocomposite electrodes have capitalized on synergistic effects, resulting in better energy storage capacity, electrical conductivity, and mechanical stability [257,258,259]. These nanotechnology advancements in electrodes have led to next-generation supercapacitors with superior energy storage, fast charge–discharge rates, and enhanced stability. As research progresses, we can anticipate even more innovative applications of nanotechnology in high-performance supercapacitor electrodes. A summary of nanomaterial application in supercapacitors is presented in Table 5.

## 4. Manufacturing Approaches for Nanomaterial Applications

**Figure 16:**
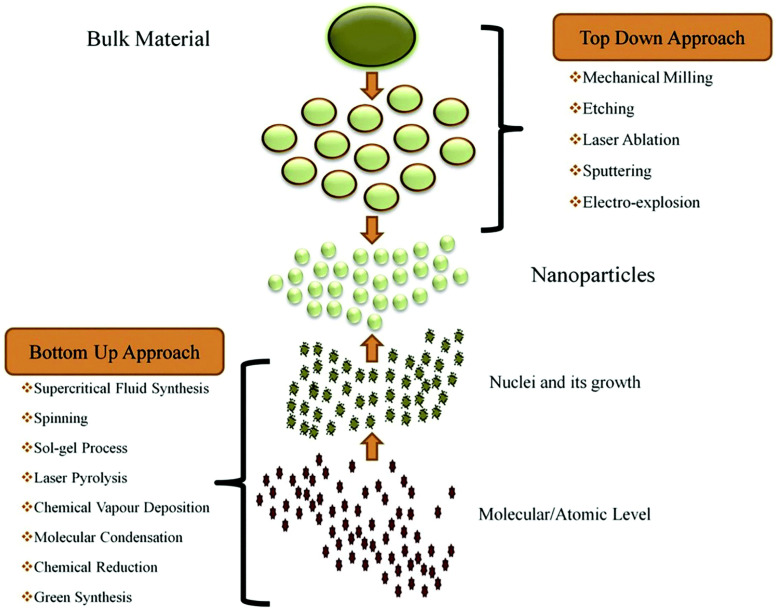
Synthesis of top–down and bottom–up approaches. © Elsevier, 2019 [271].

Nanoparticle synthesis includes a broad range of techniques, providing adaptable pathways to produce nanoparticles from diverse materials. These synthesis methods generally fall into the following two main approaches: top–down and bottom–up. Top–down methods, like ball milling, sputtering, and laser ablation, work by breaking down larger structures into nanoparticles. On the other hand, bottom–up approaches—such as sol–gel processes, chemical vapor deposition, etc.—assemble nanoparticles starting from atomic or molecular levels, offering precise control over their structure (see Figure 16). These methods greatly enhance nanotechnology’s potential by enabling the creation of nanoparticles tailored to meet specific application needs.

### 4.1. Top–Down

Top–down methods are those that begin with a macroscopic or bulk level and systematically break down materials or systems into smaller, more refined components. These approaches are particularly valuable in fields like nanotechnology, materials science, engineering, and project management. Top–down approaches are characterized by their progression from general ideas to specific details, enabling a systematic process that typically allows for precise manipulation of the final properties and characteristics of the materials or systems being created. The basic concept is shown in Figure 16. A reasonably priced technique for creating nanosized substances from large units is mechanical milling.

#### 4.1.1. Ball-Milling Method

An efficient technique for creating phase blends and a useful tool for creating nanocomposites is mechanical milling. Mechanical milling involves grinding bulk materials into nanoparticles through mechanical forces, providing control over particle size and morphology. Carbide-strengthened aluminum alloys and many additional nanocomposite substances are all made by mechanical milling [272]. Temperature is also a significant factor in mechanical milling, as the energy from ball impacts generates heat, which can influence both diffusion rates and defect formation in the powder. This heat impacts phase transitions within the material: higher temperatures tend to encourage the formation of phases requiring more atomic mobility, such as intermetallic compounds, while lower temperatures support the formation of amorphous and nanocrystalline structures. To maintain steady particle size and consistent material properties, it is essential to balance particle fracture with agglomeration throughout the milling process [272].

In mechanical milling, the process is primarily driven by the energy from collisions between milling balls and powder particles. This kinetic energy, dependent on the mass and speed of the balls, is maximized by using dense materials like steel or tungsten carbide [273]. Precise optimization of ball size and spacing is essential for efficient milling. An overly dense configuration restricts movement, while sparse distribution decreases collision frequency, slowing down the milling [274].

#### 4.1.2. Sputtering

**Figure 17:**
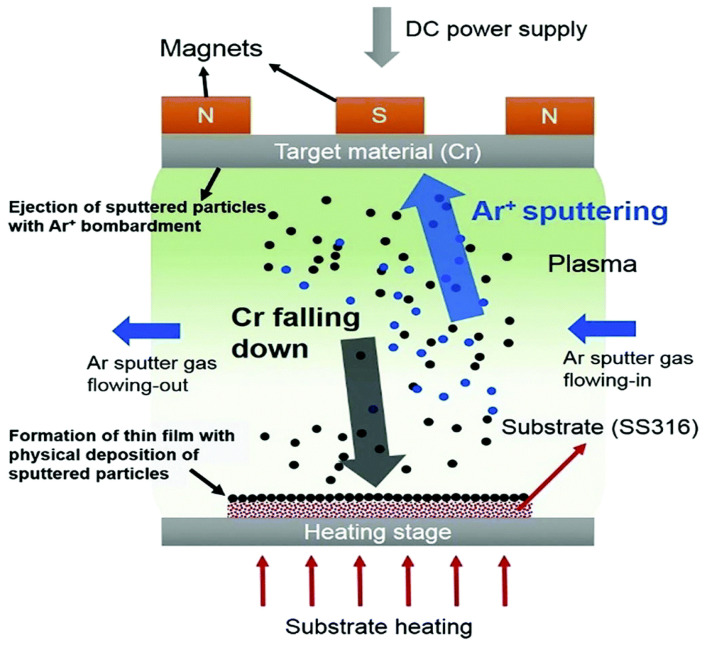
DC magnetron sputtering method. © Elsevier, 2017 [276].

Sputtering involves using high-energy particles, such as gas or plasma, to bombard solid surfaces, creating nano-atoms. It is an effective method for producing thin nanomaterial films [275]. In the deposition process, the target surface is bombarded with intense gaseous ions, causing the physical ejection of small particle clusters, depending on the ion energy (see Figure 17) [275,276]. Various techniques for sputtering include magnetron, DC diode, and radio-frequency diode sputtering [277]. During the process, sputtering gas is introduced into an evacuated chamber. The positive electrode target is subjected to high voltage, causing gas and free electrons to collide and form gas ions. The cathode target is continuously bombarded by highly accelerated positively charged ions, leading to the ejection of atoms from the target’s surface [278].

This hierarchical structure ensures that high-level goals or designs inform the development of lower-level components. In nanotechnology, several techniques exemplify top–down approaches. Lithography employs methods like photolithography and electron beam lithography to pattern materials at the nanoscale, which is crucial for microelectronics and device fabrication. Etching techniques, such as wet and dry etching, are used to selectively remove material and create nanoscale features, often following lithographic processes. Additionally, laser ablation uses focused laser beams to vaporize or ablate material, generating nanoparticles with precise characteristics. Another example is mechanical exfoliation, which physically peels layers from materials, such as obtaining graphene from graphite to create thin nanosheets [279].

In conclusion, top–down approaches provide a structured and systematic method for breaking down complex systems or materials into manageable parts. They play a crucial role in various fields, especially in nanotechnology, where precision and control at the nanoscale are essential. By starting from a broad perspective and refining to specific details, these approaches help ensure that all components work harmoniously toward achieving overarching objectives.

### 4.2. Bottom–Up Approaches

Bottom–up approaches are methodologies that begin with the fundamental building blocks or components and systematically assemble them into larger systems or structures. A key characteristic of bottom–up approaches is their focus on starting from the smallest unit or element, which is then combined to create more complex systems, allowing for emergent properties and functionalities. In nanotechnology, bottom–up methods are particularly significant for their ability to create nanoscale materials and structures with high precision and tailored properties. Material scientists often leverage biologically and naturally inspired self-assembly techniques to achieve specific nanostructures.

#### 4.2.1. Chemical Vapor Deposition (CVD)

Chemical vapor deposition (CVD) is a technique that facilitates the growth of thin films and nanostructures by depositing atoms or molecules onto a substrate in a controlled manner. This method is crucial for producing carbon-based nanomaterials. It involves a chemical reaction of vapor-phase precursors to form a thin coating on the substrate’s surface. Initially, a carbon-containing gas, such as hydrocarbons, is gradually introduced into the system. At high temperatures, the gas decomposes, releasing carbon particles that recombine on the substrate to form carbon nanotubes [280].

**Figure 18:**
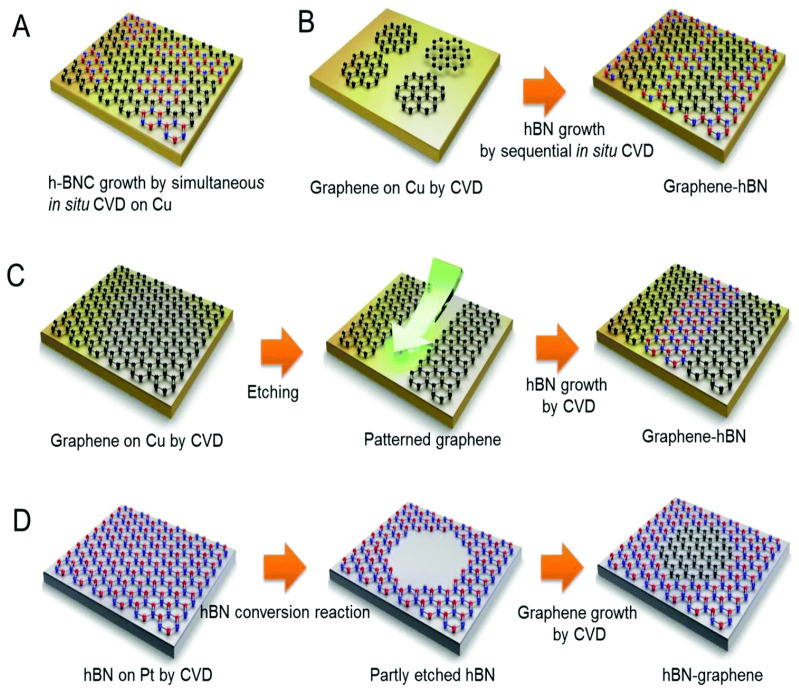
The CVD approach. (**A**) In situ CVD growth simultaneously, (**B**) in situ CVD growth sequentially, (**C**) growth of assisted lithography, and (**D**) growth of conversion. © Elsevier, 2016 [282].

CVD is a versatile method for depositing materials in their vapor phase onto a substrate, allowing for the precise fabrication of thin films and nanowires from various substances. In energy storage, CVD is essential for the uniform production of meticulously engineered nanomaterials, which significantly improve the performance of batteries and capacitors by enabling faster charge and discharge rates. CVD offers precise control over material properties and structures, enhancing efficiency and advancing energy storage solutions, thereby driving innovation in the industry [281]. The production of a two-dimensional material with CVD is shown in Figure 18.

#### 4.2.2. Sol–Gel Approach

**Figure 19:**
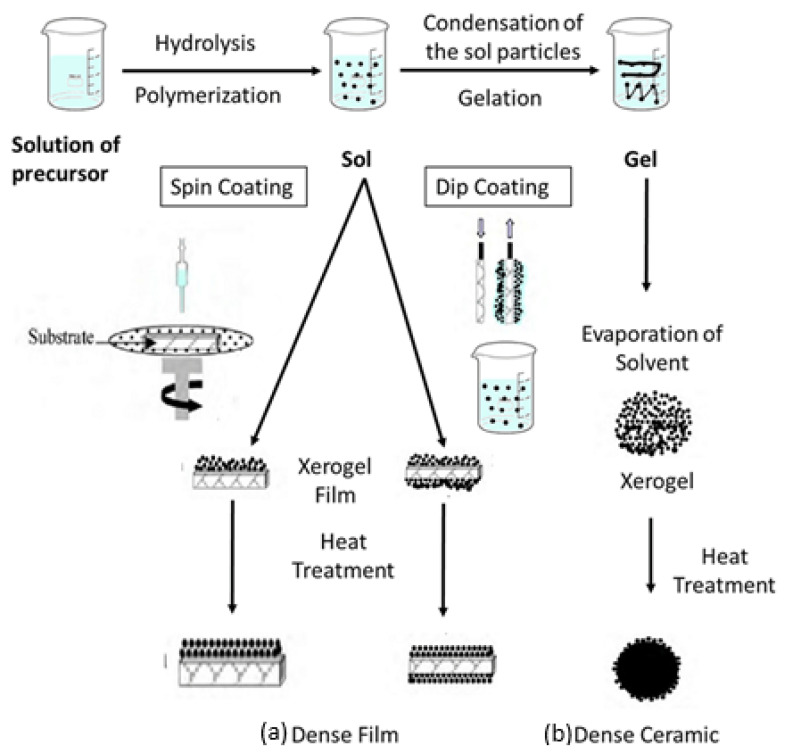
The synthesizing process of the sol–gel approach: (**a**) conversion of colloidal sol into film; (**b**) powder of colloidal gel converts into gel. © Elsevier, 2010 [286].

This method is a favored wet chemical process for creating nanotechnology. This method is used to create a wide number of excellent metal-oxide-based nanotechnologies. The reason this procedure is called the sol–gel approach is that the liquid substrate is converted into a sol during the metal-oxide nanotechnology manufacturing procedure, which is subsequently transformed into a network structure called a gel [283]. Majority of the substrates utilized in the sol–gel technique to produce nanomaterials are metallic alkoxides. Using this method to create nanotechnology entails multiple steps. The following step is condensation, which increases the solvent’s viscosity and creates pores that may emerge. Metal–hydroxo-polymer or metal–oxo-polymer production in solution is the result of hydroxo- (M–OH–M) or oxo- (M–O–M) linkages forming during the processes of condensation or polymer condensation phase [284]. The film and power formation process is shown in Figure 19.

This technique is the versatile chemical procedure for synthesizing solid materials from liquid solutions, widely used in creating nanomaterials such as metal oxides and polymers. Through controlled chemical reactions, a solution (sol) transitions gradually into a solid state (gel). This method allows accurate control over particle size, shape, and configuration, enabling researchers to develop advanced materials with enhanced energy storage capabilities. Its ability to finely tune material properties makes the sol–gel process an essential tool in modern materials science and engineering, with applications spanning catalysis, optics, and battery technologies [285].

Quantum dots, by contrast, are tiny crystals composed of several hundred atoms, with size-dependent visible wavelength spectra, making them highly effective as markers in various applications. Self-patterning generally follows two primary methods: physical and chemical. In the physical approach, straightforward growth mechanisms such as the island (Volmer–Weber) and layer-thin-island (Stranski–Krastanov) models are used. Alternatively, the chemical approach employs microemulsion techniques to form nanocrystals within colloidal suspensions, which can then be deposited onto targeted substrates. Quantum dots have promising applications in areas such as nanofluid systems, fluorescence-activated cell sorting (FACS), among others. Overall, bottom–up methods rely on naturally occurring, well-defined structures to produce advanced materials with tailored properties [272].

#### 4.2.3. Hybrid Approaches

Hybrid nanomaterials represent a major progress in nanotechnology, combining two or more different materials to leverage their unique properties and functionalities. By integrating different nanoscale components—such as metals, semiconductors, polymers, and ceramics—hybrid nanomaterials can exhibit enhanced performance characteristics that are not achievable by individual components alone. This approach opens new possibilities across different applications, such as energy storage, electronics, and medicine [287,288,289,290,291,292,293,294,295].

A significant benefit of hybrid nanomaterials lies in their capacity to combine the strengths of their individual components effectively. For instance, combining metal nanoparticles with semiconductor materials can result in improved charge separation and enhanced catalytic activity, making them particularly valuable in energy conversion functions like fuel cells. Additionally, the incorporation of polymers can provide flexibility and processability, facilitating the introduction of less heavy and durable materials. This versatility allows for the design of nanomaterials that can be suited for specialized areas, thus broadening their usability in different industries [296]. The development of hybrid nanomaterials also presents challenges that require careful consideration. Issues related to stability, biocompatibility, and scalability must be addressed to ensure that these materials can be effectively utilized in real-world applications. For instance, understanding how different materials interact at the nanoscale is important for preserving the desired characteristics over time. Moreover, regulatory considerations regarding the safety and environmental effect of hybrid nanomaterials necessitate comprehensive research to establish guidelines for their use in various applications [296].

Methods such as printing, knitting, and spray deposition are promising, while advanced techniques like 3D printing, roll-to-roll processing, solution-based self-assembly, and atomic layer deposition are becoming essential for fabricating devices using nanomaterials unsuitable for traditional slurry-based approaches [297,298,299,300,301,302,303,304]. These techniques hold significant promise for developing flexible, stretchable, and wearable energy storage systems, which are critical for the Internet of Things (IoT) and other emerging technologies [305,306,307,308,309,310,311,312,313].

Looking ahead, the future of hybrid nanomaterials is bright, with ongoing research focused on optimizing their properties and expanding their applications. Innovations in synthesis methods, such as bottom–up and top–down approaches, are paving the way for more efficient and scalable production processes. As we continue to explore the potential of hybrid nanomaterials, their integration into everyday products and technologies is likely to revolutionize fields ranging from energy to healthcare, ultimately contributing to a more sustainable and advanced technological landscape [314].

## 5. Challenges and Perspectives

Nanoparticles’ large surface area can lead to reactions with electrolytes, particularly causing irreversibility during the first cycle. Regarding environmental impact, nanomaterials can penetrate cells and tissues, potentially harming DNA, proteins, and membranes. To enhance safety and stability, encapsulation may be required. Long-term stability is crucial, as the materials must remain durable over extended periods, and effective purification is necessary to ensure nanoparticle quality. Nanoparticles interact uniquely with electrolytes, which can influence their stability and introduce potential safety risks. To fully exploit the prospect of nanoparticles in advanced energy storage systems, it is essential to understand the intricate relationship between electrochemical behavior, material properties, and physical principles.

### 5.1. Electrode Rupture

Conventional intercalation-related electrodes can vary in volume, which is less than 10% when lithium is added to or removed from a system. Newer electrode materials typically experience bigger volume fluctuations due to large amounts of lithium intake. Since the 1990s, the greater-capacity uses have been constrained by the greater fluctuation of volume during loading or unloading incidents. For example, the volume change in some lithium alloy anodes such as Si, Ge, and Sn is 420%, 260%, and 300%, respectively. These metals have volume fluctuations that are mostly larger than 10% of the anode of the graphite. The volume change of the metal lithium anode can be virtually infinite because of their environment without the host. This dramatic volume change can probably degrade active materials and electrodes during cycling, resulting in degrading the cycle life [315].

### 5.2. Electron Transportation in Particles

A better efficiency of a battery relies on a higher ratio of electrons or ions moving in each particle. For activating insulating substances, strong electron transmission routes and shorter ion movement distances can assist to accomplish a better-performing holding. Nanoparticles (independent particles) can add or remove lithium ions from their surroundings and move electrons compared to microparticles due to the shorter travel times. Two techniques are utilized to raise the conductance of each particle independently. These are coating active particles with conduction layers and putting particles in conductive material [315].

### 5.3. Low Coulombic Efficiency

The development of nanomaterials for next-generation LiBs has garnered notable global interest due to their probable enhanced storage capacity, as their electrochemical properties are highly dependent on size and shape. Over the past two decades, nanotechnology has been a major focus in the battery community. However, a critical yet often overlooked challenge is the low energy density of nanomaterials due to their low volumetric density. When it comes to the SEI layer’s consumption of electrolyte and lithium on the cathode surface during the battery’s cycle, nanoparticle-type electrodes use more electrode and lithium than micro structured material because of the greater contact with the electrode and electrolyte surface region, resulting in a poor starting Coulombic performance. For instance, graphene, a widely researched nanomaterial, has a tap density of just 0.03 g/cc, compared to 1.3 g/cc for MCMB graphite powder—a 40-fold volumetric difference for the same weight. Additional issues include poor electrical performance caused by resistance between particles and low Coulombic efficiency, resulting from side reactions between the electrode and electrolyte resulting from the the large surface region of nanoscale substances [315].

**Figure 20:**
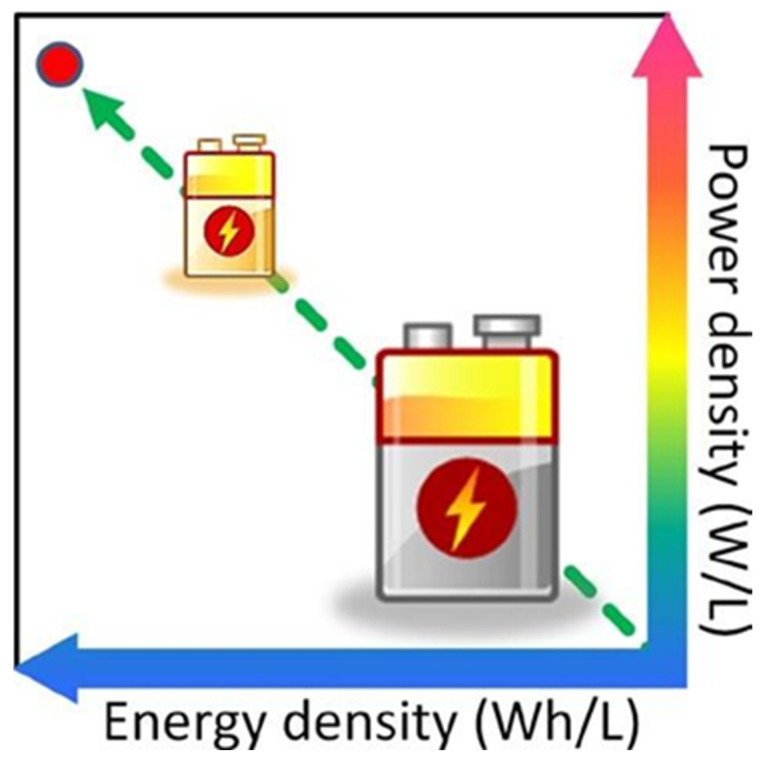
Evidence of later generations’ LiBs ought to be lightweight and slim without sacrificing power or energy [322]. © Energy Science & Engineering, 2015.

To improve volumetric density, micromaterials of nanoparticle aggregates have been developed for some time [316,317,318,319,320,321]. For instance, microscale cubes of Zn_2_SnO_4_ and Sn@C nanoparticle aggregates show a tap density of 0.98 g/cc, which is approximately eight times greater than that of commercial P25 TiO_2_ nanoparticles [316]. Furthermore, micromaterials demonstrate electrochemical performance comparable to nanomaterials. Since devices like cell phones and electric vehicles have limited space for batteries, future electrode materials will need high tap density. Hence, it is anticipated that future LiBs will utilize micromaterials with nanomaterial-like properties to address volumetric density requirements [322]. Figure 20 illustrates the relation between energy density and power density, but size should be reduced in the long run.

There has been significant progress in the development of new battery technologies, such as potassium-ion batteries (PIBs), which studies have proved to be largely superior to LiBs in terms of both economic and functional properties. PIBs leverage potassium, which is significantly more abundant and less expensive than lithium, thus reducing overall production costs and enhancing accessibility for large-scale applications [323,324,325]. In terms of energy density, potassium’s standard reduction potential is comparable to lithium’s, which makes it a viable alternative for applications requiring high energy storage [324]. Zhang et al. [324] in their study highlight that the adoption of effective design strategies in PIBs such as heteroatom doping [326], electrolyte optimization [327], and using composite structures in the design of electrodes [328] can significantly address a major challenge faced by the current energy storage technologies.

Lastly, while research into nanomaterials for energy storage is expanding, there remains a lack of comprehensive studies that explore the continued stability of these materials under practical working environments. Continued investment in research and development is essential to address these challenges effectively. Despite these obstacles, ongoing research and innovation in nanotechnology offer promising solutions for enhancing energy storage systems [329,330,331,332,333]. By concentrating on scalable manufacturing methods, cost-reduction strategies, and environmental safety, the potential of nanomaterials can be harnessed to improve energy storage solutions, ultimately contributing to a more sustainable energy future [334].

## 6. Economic Analysis

Nanoparticles offer a cost-effective solution for improving energy storage. Their nanoscale size increases surface area, enhancing battery and capacitor performance by enabling higher energy density, longer lifespans, and reduced reliance on costly materials. Additionally, nanoparticles can be incorporated into advanced insulating materials to increase energy efficiency in buildings, decreasing heating and cooling costs. Their affordability and versatility make them a cost-effective and sustainable solution for transforming energy storage [335].

Cost-effectiveness is a concern due to the high expenses associated with large-scale nanomaterial synthesis. Recent advancements in nanomaterials, particularly in the development of aerographene and graphene aerogel, are revolutionizing energy-related technologies. These materials, especially when fabricated using cutting-edge 3D and 4D printing techniques, are finding diverse applications across various industries. Graphene aerogels are especially promising due to their highly permeable and tiered structure, which allows for rapid electron and ion transport, improved chemical and physical stability, and exceptional cycling performance. These qualities make graphene aerogels particularly suitable for energy systems, including supercapacitors, fuel cells, and solar cells. The increasing demand for efficient energy storage solutions is a significant driver of growth in the aerographene and graphene aerogel markets [336].

**Figure 21:**
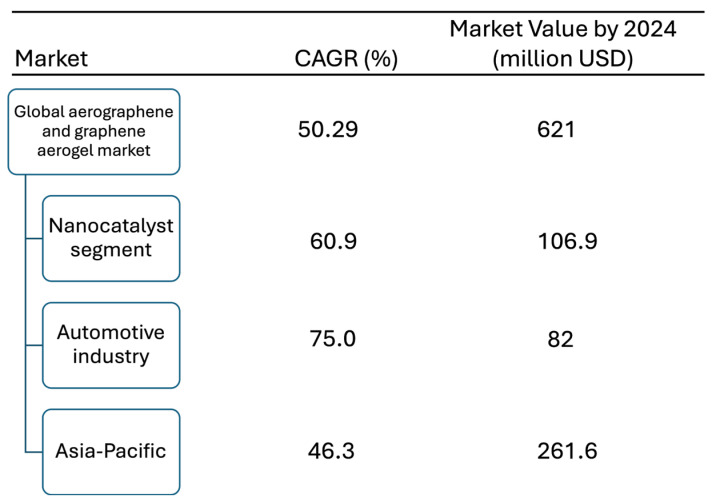
Economic impact of the advancement of nanomaterials in energy-related technologies [336].

The global market for aerographene and graphene aerogels was valued at USD 51.0 million in 2018 and is projected to grow to USD 621.0 million by 2024, representing a remarkable compound annual growth rate (CAGR) of 50.29%. Within this market, the nano-catalyst segment is expected to experience the fastest growth, with a projected CAGR of 60.9%, expanding from USD 5.2 million in 2018 to USD 106.9 million by 2024. Nano-catalysts are critical for enhancing the efficiency of chemical reactions in processes such as fuel cells and hydrogen production, underlining the growing importance of these materials in the energy sector [336]. Figure 21 summarizes the economic impact of the aerographene across the globe.

In addition, the automotive industry is forecasted to see rapid growth in the application of aerographene and graphene aerogels, with a predicted CAGR of 75.0% and a market value expected to reach USD 82.0 million by 2024. This surge is driven by the necessity for light and high-performance materials in electric vehicles (EVs), where aerogels provide excellent thermal insulation, energy storage, and mechanical properties. Their integration into EV batteries and their role in improving vehicle efficiency and reducing emissions highlight their potential to transform the automotive sector.

Regionally, the Asia–Pacific held the principal share (52.2%) of the global aerographene and graphene aerogel market in 2018 and is projected to reach USD 261.6 million by 2024, with a CAGR of 46.3%. This growth is attributed to strong government policies supporting energy efficiency and renewable energy solutions, particularly in China, Japan, and South Korea. These countries are leading in the development and adoption of nanotechnology for energy storage, with a focus on achieving energy independence and reducing reliance on fossil fuels [336].

In Europe, countries like Germany and the United Kingdom are also investing heavily in nanotechnology for energy applications, driven by the continent’s focus on clean energy and stringent environmental regulations. Africa, Latin America, and the Middle East are likely to see substantial growth in the graphene aerogel market, with Brazil leading Latin America’s efforts despite economic challenges stemming from the COVID-19 pandemic. A notable frontier in aerogel technology is the development of 3D- and 4D-printed graphene aerogels. These printed aerogels exhibit superior mechanical properties, making them resist repeated compression, making them ideal for use in flexible batteries and energy storage devices. They also show potential in energy conversion, catalysis, and separation technologies, with their large internal surface area being particularly advantageous.

The global aerographene and graphene aerogel market is highly competitive, with key players like Aerogel Technologies LLC, American Elements, and Graphene 3D Lab Inc. leading the way. These companies are heavily invested in research and development to push the boundaries of nanomaterial applications. As the market continues to grow, these materials will become increasingly integral to future energy storage and conversion technologies [305]. An essential focus area deserving greater attention is the techno-economic analysis (TEA) of LiBs. TEA plays a critical role in assessing whether a newly developed LiB methodology is both technically viable and economically suitable for wholesale production. By integrating TEA with life cycle assessment (LCA) studies, researchers can advance next-generation Li-ion batteries that strike an optimal balance among social, environmental, and economic impacts [322].

## 7. Sustainability and Environmental Concerns

As the demand for compact and lightweight portable electronics grows, efficient energy storage solutions are required with high power, energy density, and longevity, which has become critical. These energy storage systems are vital for promoting sustainable energy innovations. Electrochemical devices provide numerous advantages, such as affordability, durability, high energy and power densities, reversibility, and environmentally friendly performance.

The environmental impact of producing, using, and disposing of nanoparticles must be considered when developing sustainable energy storage technologies. Nanoparticles, integral to devices like supercapacitors and advanced batteries, are often derived from materials such as lithium, cobalt, and other rare elements. However, their production processes are resource-intensive and energy-demanding, contributing significantly to greenhouse gas emissions. Moreover, the extraction and processing of these resources can lead to environmental degradation and habitat destruction. During the usage phase, it is crucial to address worries regarding potential nano-atom emissions and their impact on ecosystems, along with the energy efficiency and environmental footprint of the energy storage systems they support. To prevent pollution of landfills and ecosystems, the disposal of these substances must involve responsible recycling and disposal methods. Addressing these environmental concerns is essential to ensuring that sustainable energy storage technologies genuinely contribute to a better future [337].

Creating electrode materials with a minimal carbon footprint—also known as “green batteries”—remains a difficult task. Future LiBs should ideally employ aqueous electrolytes and organic or inorganic electrodes that are biologically formed. Biomasses are a source of silicon and carbon. There was a lot of interest in the recent initiatives to investigate virus-assisted production of electrode materials for LiBs [338]. Comprehensively investigating synthesis at room temperature with biological templates, such as synthetically produced viruses, will be attractive. For durable Li-ion batteries, organic electrodes that are difficult for electrolytes to dissolve can be further improved [339]. Therefore, concentrating on “sustainable” and “green” Li-ion batteries could be one topic of future research.

The LCA of technologies should also be included in future battery research to determine whether the batteries are actually green. From an LCA standpoint, more research is still needed on the current materials used in commercial LiBs. Cathode substances are mostly LiCoO_2_, LiMn_2_O_4_, LiNiO_2_, or LiFePO_4_; anode materials are graphite and Li_4_Ti_5_O_12_; electrolytes and salts are ethylene carbonate, diethyl carbonate, LiPF_6_, LiBF_4_, and LiClO_4_; and separators are polypropylene (PP) and polyethylene (PE). The effects of the manufacture, usage, and disposal of next-generation LiBs on the environment are not well understood [340]. When Li-ion batteries for electric cars are produced in significant quantities, hazardous waste and locations will be produced. It might raise energy costs close to factories, lower air quality near processing plants, and lower agricultural output close to mine sites. In addition, it might be necessary to consume more fossil fuels to meet manufacturing demand for Li-ion battery production. Their lifespan, from the acquisition of raw minerals to manufacturing, consumption, expiry, recycling, and final disposal, LCAs may outline all environmental factors and potential environmental implications. To create genuinely “green” Li-ion batteries in the future, which supports sustainability, LCA is a crucial tool [322]. Statistical data indicate that in 2020, approximately 25 billion lithium-ion batteries (LiBs), weighing around 500,000 tons, were discarded. Improper handling of these recycled LiBs poses a significant risk of releasing harmful contaminants [341].

One critical aspect of responsible nanotechnology integration in agriculture is the determination of safe nanoparticle dosage levels. To achieve this, concentration-dependent studies should be conducted in natural soil environments to identify both effective and non-toxic doses of nanoparticles. Such studies would provide valuable insights into the safe usage of nanoparticles, helping to ensure that they do not pose a risk to plants, soil organisms, or the broader ecosystem [342]. Additionally, understanding the transgenerational effects and the movement of nanoparticles through the trophic chain in plants and other organisms is essential for evaluating long-term nanotoxicity. These studies will allow researchers to assess how nanoparticles may accumulate or transfer through different levels of the food chain, contributing to more comprehensive safety evaluations [343].

## 8. Conclusions

This paper highlights the transformative potential of nanotechnology in enhancing energy storage systems, particularly in the context of the ever-increasing global energy demand. It emphasizes that manipulating materials at the nanoscale can lead to significant improvements in the performance of energy storage devices such as capacitors and batteries, including lithium-ion, sodium–sulfur, and redox flow batteries. The unique properties of nanomaterials, including their electrical, mechanical, and interfacial characteristics, make them more efficient compared to conventional materials. This efficiency is largely attributed to the greater contact area between electrodes and electrolytes, which facilitates better site reactions, ultimately leading to enhanced energy storage performance.

The paper also delves into various manufacturing methods for nanomaterials, categorizing them into top–down, bottom–up, and hybrid approaches. Each of these methods presents its own set of advantages and challenges, particularly concerning scalability and cost-effectiveness. For instance, bottom–up approaches utilize self-assembly techniques to create nanostructures, while top–down methods involve breaking down bulk materials into nanoscale components. The hybrid approaches combine the strengths of both methods, allowing for the development of advanced materials that can meet the demands of modern energy storage systems.

Despite the promising outlook for nanotechnology in energy storage, the review identifies several challenges that need to be addressed. Issues related to scalability, cost-effectiveness, and environmental concerns associated with the production and use of nanomaterials are significant barriers to widespread adoption. The economic analysis presented in the paper indicates that the Asia–Pacific region is a major player in the nanomaterials market for energy storage, driven by strong government policies and a focus on energy efficiency and renewable energy solutions. This regional emphasis highlights the importance of supportive policies in fostering innovation and development in this field. Furthermore, the review addresses the sustainability and environmental implications of using nanomaterials in battery production. While the benefits of nanomaterials are clear, the paper stresses the need for careful consideration of their environmental impacts. This includes evaluating the lifecycle of nanomaterials, from production to disposal, to ensure that their use contributes positively to sustainability goals.

Advancing energy storage technology is essential for integrating renewable energy sources into existing systems, and the demand for efficient energy storage solutions is expected to grow, making research in this area increasingly important. This is critical to overcoming existing challenges, improving performance, advancing manufacturing techniques, addressing environmental concerns, and enhancing sustainability in energy storage systems. This ongoing effort will ultimately contribute to a more efficient and sustainable energy future.

## Figures and Tables

**Table 1:** Comparison of some negative electrode materials [22,24,25,26,27] © Royal Society of Chemistry, 2017.

Material	Advantages	Disadvantages
Carbon	High electronic conductivity	Low specific capacity
	Nice hierarchical structure	Low-rate capacity
	Abundant and low-cost resources	Safety issues
Alloys	High specific capacity (400–2300 mA h g^−1^)	Low electronic conductivity
	Good security	Large volume change (100%)
Transition metal oxides	High specific capacity (600–1000 mA h g^−1^)	Low coulombic efficiency
	Nice stability	Large potential hysteresis
Silicon	Highest specific capacity (3579 mA h g^−1^)	Large volume change (>300%)
	Rich, low-cost, a from a clean resource	

**Table 2:** Summary of electrochemical performance of negative electrode nanomaterials in LiBs.

	Material	Electrochemical Performance	Ref.
Insertion	Carbon-based	Significant reversible cycles, chemical stability, electrochemical stability, thermal stability.	[28]
	Carbon-based (with coating)	Improved performance due to thinner and denser SEI film	[29]
	Titanium-based	Minor safety issues, less toxicity, little volume change (2–3%), extended cycle life	[30,31,32,33]
	Carbon Nanotubes (CNTs)	- High conductivity and stability - Can absorb Li-ions on both internal and external surfaces	[21,36,38]
	Graphene	High conductivity, structural flexibility, higher charge mobility, lightweight, good surface area	[46,47]
Conversion	Iron Oxides (Fe_2_O_3_, Fe_3_O_4_)	High theoretical capacity (~1000 mAh/g), non-toxicity, excellent reversible capacities	[62,66,68,69,70,71]
	Cobalt Oxides (CoO, Co_3_O_4_)	High theoretical capacity (Co_3_O_4_: 890 mAh/g, CoO: 715 mAh/g), good capacity retention	[84,85,86,87,88,89]
Alloys	Silicon (Si)	- High theoretical specific capacity (4211 mAh/g) - High volumetric capacity (9786 mAh/cm^3^) - Abundant and environmentally friendly	[62,90,91,92,93,94,95,96,97]
	Silicon Nanowires	- Can withstand greater volume changes due to their nanoscale size - Direct growth on the current collector allows for quick charge transfer	[22]
	Germanium (Ge)	- High electrical conductivity (10,000 times greater than Si) - High theoretical capacity (1623 mAh/g) - High Li-ion diffusion rate	[62,92,100,101,102]
	Germanium Nanowires	- Can effectively suppress volume changes due to their nanoscale size - Enhanced electrochemical performance with high reversible capacity and cycling stability	[101,103,104,105]

**Table 3:** Summary of electrochemical performance of nanomaterials in Na-S batteries.

Component	Nanomaterials	Electrochemical Performance
Electrolyte	Solid-state electrolytes (e.g., FSA-Na)	These membranes serve as both electrolyte and separator, enhancing stability and controlling the shuttle effect [167].
Separator	Solid-state electrolyte membranes	These membranes help prevent polysulfide shuttling and improve safety in sodium–sulfur batteries [167].
Cathode	Vanadium carbide nanoparticles in carbon nanofibers (VC-CNFs)	These materials enhance electrochemical performance, acting as chemical trappers and electrocatalysts to mitigate the shuttle effect and improve reaction kinetics [165].
	Nanocomposite catalytic cathodes	Incorporation of various nanomaterials (metal oxides, sulfides, single atoms) into porous carbon hosts accelerates the conversion of sulfur species and enhances reaction kinetics [161,162].
Anode	Nanostructured host materials	These materials help mitigate dendrite growth and volume expansion during cycling of sodium metal anodes [152].
	Transition metal nanoparticles or single atoms	These enhance sodiophilicity and improve the stability of the interphase of the solid electrolyte [156,157,158].

**Table 4:** Summary of electrochemical performance of nanomaterials in redox flow batteries.

Component	Nanomaterials	Key Features	Electrochemical Performance
Electrodes	Carbon-based nanomaterials [208,209,210,211]	High surface area	Enhanced mass transport
		Superior conductivity	Improved charge transfer
		Chemical stability	Increased electrocatalytic activity, leading to better cell performance
	Metal nanoparticles (e.g., platinum, palladium, and gold) [184,185]	High conductivity	Improved efficiency
		Electrocatalytic activity	Enhanced durability
			High cost and side reactions are concerns
	Metal oxide nanoparticles (e.g., CeO_2_ and MnO_2_) [193,194,195,196,197]	Economical	Enhanced reaction kinetics
		High catalytic activity	Bifunctional catalytic behavior for both positive and negative reactions
Electrolytes	Carbon-based nanofluids (e.g., incorporating graphene and carbon nanotubes) [216,217,218]	Large surface area	Improved conductivity
		Porous configuration	Enhanced electrochemical reaction kinetics
		Good conductivity	
	Metal-based nanoparticles [215]	Rapid electron migration	Enhanced electron transport within the electrolyte
			Improved battery performance
	Suspended nanofluids [214]	Intermediate properties between liquids and solids	Improved electrochemical reaction kinetics
			Better ion transport
Separators	Organic nanomaterials (e.g., poly(4-vinyl pyridine), polypyrroles, and polyaniline) [230,231,232]	Enhanced selectivity	Reduced crossover of active species
		Improved ionic conductivity	Better overall battery performance
	Inorganic nanoparticles (e.g., SiO_2_ and ZrP) [230,231,232]	Modification of the nafion matrix	Hinders passage of larger active species
		Creation of tortuous paths for ion transport	Increases efficiency of ion transport

**Table 5:** Summary of electrochemical performance of nanomaterials in supercapacitors.

Component	Nanomaterials	Electrochemical Performance
Electrodes	Carbon-based nanomaterials	Improve conductivity and surface area, resulting in increased capacity and quicker rates of charging and discharging [260].
	Metal oxides (e.g., RuO_2_ and MnO_2_)	Metal oxides, with their high capacitance and stability, are ideal for high-performance supercapacitors [261].
	Conductive polymers (e.g., polyaniline and polypyrrole)	Provide adequate electrical conductivity and easy processing, contributing to the overall performance of the electrode [262,263].
	MOFs and COFs	Tunable chemical composition, excellent stability, enhanced conductivity, high surface area, and porosity [250,251].
Electrolytes	Ionic liquids	Ionic liquids have a wide electrochemical window and high ionic conductivity, making them ideal for supercapacitor applications [264].
	Gel electrolytes	Gel electrolytes incorporate the benefits of solid and liquid electrolytes, providing excellent ionic conductivity and enhanced safety [265,266].
Separators	Nanofibers (e.g., polyvinylidene fluoride (PVDF) nanofibers)	Possess exceptional mechanical strength and can enhance the ionic conductivity of the separator [267,268].
	Porous membranes	Allow for efficient ion transport while preventing electrical short circuits between the electrodes [269,270].

## Data Availability

The data presented in this study are available upon request from the correspondence author.
